# 5,7,4′‐Trimethoxyflavone triggers cancer cell PD‐L1 ubiquitin–proteasome degradation and facilitates antitumor immunity by targeting HRD1

**DOI:** 10.1002/mco2.611

**Published:** 2024-06-27

**Authors:** Jianhua Xia, Mengting Xu, Hongmei Hu, Qing Zhang, Dianping Yu, Minchen Cai, Xiangxin Geng, Hongwei Zhang, Yanyan Zhang, Mengmeng Guo, Dong Lu, Hanchi Xu, Linyang Li, Xing Zhang, Qun Wang, Sanhong Liu, Weidong Zhang

**Affiliations:** ^1^ Shanghai Frontiers Science Center of TCM Chemical Biology Institute of Interdisciplinary Integrative Medicine Research Shanghai University of Traditional Chinese Medicine Shanghai China; ^2^ Department of Phytochemistry School of Pharmacy Second Military Medical University Shanghai China; ^3^ Institute of Medicinal Plant Development Chinese Academy of Medical Sciences and Peking Union Medical College Beijing China; ^4^ The Research Center for Traditional Chinese Medicine Shanghai Institute of Infectious Diseases and Biosafety Institute of Interdisciplinary Integrative Medicine Research Shanghai University of Traditional Chinese Medicine Shanghai China

**Keywords:** 5,7,4′‐trimethoxyflavone, colorectal cancer, HRD1, PD‐L1

## Abstract

Targeting the programmed cell death 1/programmed cell death ligand 1 (PD‐1/PD‐L1) pathway has been identified as a successful approach for tumor immunotherapy. Here, we identified that the small molecule 5,7,4′‐trimethoxyflavone (TF) from *Kaempferia parviflora* Wall reduces PD‐L1 expression in colorectal cancer cells and enhances the killing of tumor cells by T cells. Mechanistically, TF targets and stabilizes the ubiquitin ligase HMG‐CoA reductase degradation protein 1 (HRD1), thereby increasing the ubiquitination of PD‐L1 and promoting its degradation through the proteasome pathway. In mouse MC38 xenograft tumors, TF can activate tumor‐infiltrating T‐cell immunity and reduce the immunosuppressive infiltration of myeloid‐derived suppressor cells and regulatory T cells, thus exerting antitumor effects. Moreover, TF synergistically exerts antitumor immunity with CTLA‐4 antibody. This study provides new insights into the antitumor mechanism of TF and suggests that it may be a promising small molecule immune checkpoint modulator for cancer therapy.

## Graphical abstract

A brief description of the role of TF in promoting PD‐L1 degradation in colorectal cancer (CRC). TF binds specifically to and stably expresses HRD1, thereby increasing the ubiquitination of PD‐L1 and leading to its degradation via the proteasome, which enhances T cell activity and activates the tumor immune microenvironment.

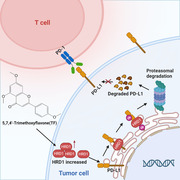

## 1 INTRODUCTION

Colorectal cancer (CRC) is a common prevalent malignancy of the gastrointestinal digestive system. Current treatment relies mainly on traditional surgical resection, but most treated patients still experience relapse and metastasis, and the tumor cannot be completely eradicated.^1^, ^2^, ^3^ Immunotherapy has brought new hope for tumor treatment because of its ability to regulate the immune microenvironment of solid tumors, such as CRC.^4^, ^5^ Infiltrating T cells in the tumor microenvironment exhibit high PD‐1 protein expression, while tumor cells concurrently overexpress the PD‐1 ligands PD‐L1 and PD‐L2, leading to continuous activation of the PD‐1 pathway, which suppresses T cell function and impairs their ability to eliminate tumor cells. PD‐1 antibodies can block this pathway, partially restoring the function of T cells and allowing these cells to continue killing tumor cells.^6^, ^7^, ^8^ Currently, immunotherapy has shown impressive efficacy in the treatment of multiple malignancies, including advanced CRC, through immune checkpoint blockade with antibodies targeting the PD‐1/PD‐L1 pathway.^9^, ^10^

Despite considerable progress has been made in the treatment of cancers highly expressing PD‐L1, such as lung cancer, melanoma, and CRC, where some cancer patients have experienced substantial improvements in survival and some have even achieved complete remission, the tumor response rate to PD‐1/PD‐L1 antibody treatments remains inadequate. For instance, the majority of patients have a low response rate to PD‐1/PD‐L1 antibody therapies and suffer from side effects.^11^, ^12^ The pharmacokinetic characteristics, tumor tissue permeability, and lower production cost of small molecule immune checkpoint blockers make them superior to PD‐1/L1 antibodies with a molecular weight exceeding 141,000 Da. Therefore, identifying drugs that can downregulate PD‐L1 expression in tumor cells may be an effective tumor treatment strategy.

The regulation of protein stability is significantly influenced by the ubiquitin–proteasome system.^13^ Ubiquitination is the process in which enzymes catalyze the modification of target proteins by ubiquitination, leading to their degradation, in which the E3 ubiquitin ligase determines the specific recognition of the target protein.^14^, ^15^ Previous studies have shown that PD‐L1 can be ubiquitinated and degraded through E3 ubiquitin ligases such as STUB1, SPOP, HRD1, MARCH8, A20, and ARIH1.^16^, ^17^, ^18^, ^19^, ^20^, ^21^, ^22^ In contrast to E3 ubiquitin ligases, deubiquitinating enzymes can remove the ubiquitin chain, reverse ubiquitination, and increase the stability of the substrate protein.^14^, ^23^ For instance, USP22, OTUB1, and CSN5 can prevent PD‐L1 on tumor cells from being ubiquitinated and degraded, which suppresses immune cells activity in the tumor microenvironment and enables cancer cells immune escape.^24^, ^25^, ^26^ Consequently, understanding how ubiquitin–proteasomes degrade targets will be useful for improving PD‐L1 immunotherapy.

HRD1 is an E3 ligase enzyme essential for disease progression because it is responsible for degrading improperly folded proteins and maintaining protein stability through ubiquitin ligation.^27^, ^28^, ^29^ P53, a tumor suppressor, can be negatively regulated by HRD1, which can also increase the ubiquitination and degradation of p53.^30^ Therefore, HRD1 dysregulation can inhibit two different apoptotic pathways induced by p53‐dependent endoplasmic reticulum stress.^31^, ^32^ HRD1 also promotes PD‐L1 expression in hepatocellular carcinoma cells by mediating the degradation of FoxO1, which in turn leads to immune evasion, cell proliferation, and metastasis.^33^ Taken together, these results indicate that HRD1 is a potential target for immune checkpoint therapy (ICB).

5,7,4′‐Trimethoxyflavone (TF) is derived from the perennial herb *Kaempferia parviflora* Wall. ex Baker in the ginger family.^34^ It has been shown to have anticholinesterase, antibacterial, anti‐inflammatory, antiaging, treatment of coronary heart disease, and neuroprotective effects.^35^, ^36^, ^37^ However, no study has investigated whether TF can play an antitumor role as an immunomodulator. In this study, we screened a library of compounds containing 96 small molecules from natural compounds and found that TF could significantly inhibit PD‐L1 expression in CRC cells. Our studies showed that TF can target HRD1 to promote the ubiquitination and degradation of PD‐L1, thereby enhancing the anti‐CRC immune response.

## 2 RESULTS

### 2.1 TF negatively regulates the expression of PD‐L1 in CRC cells

We initially screened 96 small molecules sourced from a natural compound library for their capacity to decrease PD‐L1 expression in RKO CRC cells. A brief outline of the process of screening for drugs that reduce the expression of PD‐L1 in RKO cells (Figure S1). The test compounds (20 µM) were coincubated with RKO cells for 24 h, after which the PD‐L1 levels were assessed using Western blotting. Compounds capable of reducing total PD‐L1 protein expression were further screened by flow cytometry to determine their ability to downregulate the membrane PD‐L1 of RKO cells. Among the screened compounds, only TF reduced the expression of PD‐L1 on the cell membrane of RKO cells. The results of the compound screening are presented as a heatmap in the screening flowchart, as detailed in Figure S1.

**FIGURE 1:**
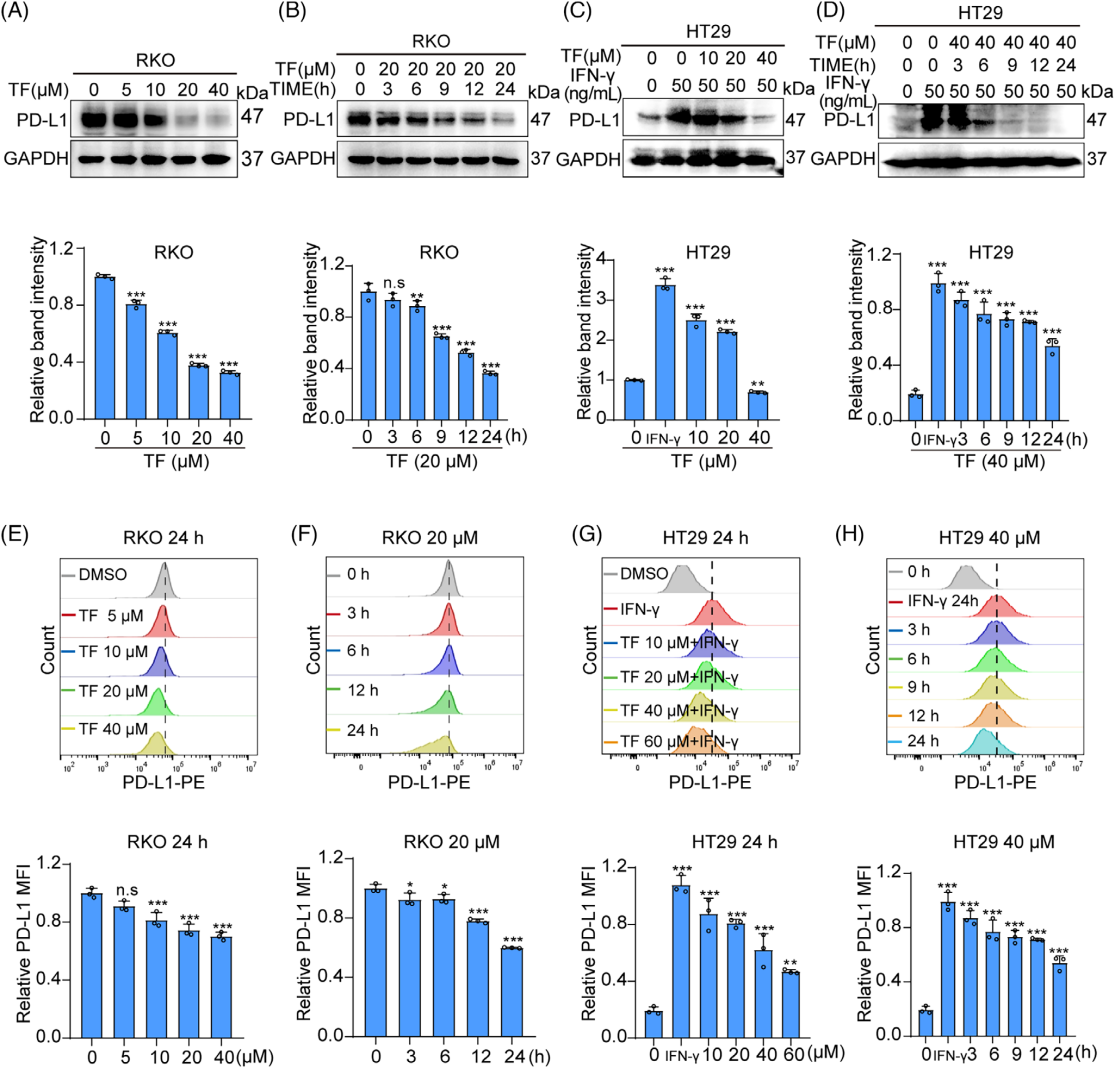
The impact of TF on PD‐L1 degradation in CRC cells. (A and B) RKO cells were exposed to varying concentrations of TF for 24 h or 20 µM TF for different durations, followed by immunoblotting (IB) analysis of total PD‐L1 protein. Quantitative IB analysis results are shown below. (C and D) HT29 cells were treated with various concentrations of TF for 24 h or 40 µM TF for different durations, and subsequent IB analysis of total PD‐L1 protein and the corresponding quantitative results are provided below. (E and F) Flow cytometry analysis of PD‐L1 membrane proteins in RKO cells after exposure to different concentrations of TF for 24 h or 20 µM TF for various durations, with quantitative flow cytometry analysis results shown below. (G and H) Flow cytometry analysis of PD‐L1 membrane proteins in HT29 cells following treatment with different TF concentrations for 24 h or 40 µM TF for various durations, accompanied by quantitative flow cytometry analysis results below. Data are presented as mean ± standard error of the mean (SEM). Statistical significance is denoted as *p < 0.05, **p < 0.01, ***p < 0.001.

Using immunoblot (IB) experiments, we found that TF decreases PD‐L1 expression in a concentration‐ and time‐dependent manner in RKO and HT29 cells (Figure 1A–D). Furthermore, flow cytometry was used to test the influence of TF on PD‐L1 expression on the membranes of CRC cells. As shown in Figure 1E–H, we also observed a concentration‐ and time‐dependent decrease in PD‐L1 expression in RKO and HT29 cell membranes, which was also confirmed via immunofluorescence (Figure S2A–D). In addition, the results of the EdU and Cell Counting Kit 8 (CCK8) assays showed that TF treatment had little inhibitory effect on RKO cells or HT29 cells in the 0–40 µM range (Figure S2E–H). In summary, our study suggested that TF negatively modulates the levels of PD‐L1 in CRC cells while exhibiting minimal cytotoxic effects.

### 2.2 TF restores the ability of T cells to kill cancer cells in vitro

**FIGURE 2:**
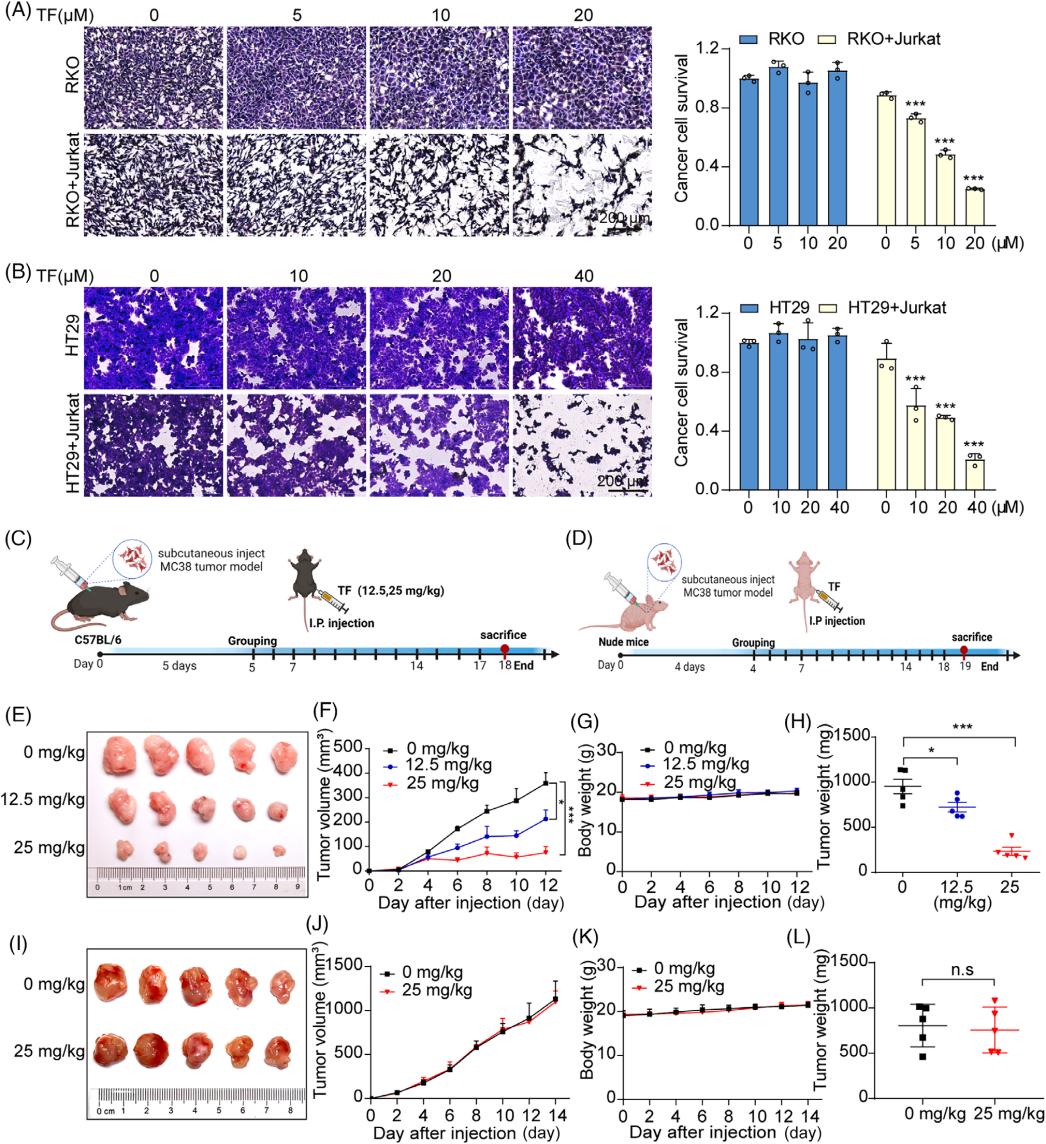
TF enhances T cell killing capacity and mediates T cell‐dependent antitumor effects in vivo. (A and B) Jurkat cells were cocultivated with RKO and HT29 cells and exposed to varying concentrations of TF for 24 h. The viability of the tumor cells was evaluated through crystal violet staining, and the results of the quantitative analysis are displayed on the right. (C) Experimental procedures for animal experiments in C57BL/6J (female) mice (i.p. intraperitoneal injection). (D) Experimental procedures for animal experiments in nude (female) mice (i.p. intraperitoneal injection). (E) Illustrative depiction of solid tumors representative of different treatment groups in C57BL/6J mice. (F) Tumor growth curves illustrating the progression in C57BL/6J mice across different treatment groups. (G) Body weight trends of C57BL/6J mice subjected to various treatment regimens. (H) Comparative analysis of tumor weights among C57BL/6J mice receiving different treatments. (I) Graphical representation of representative solid tumors observed in nude mice across different treatment groups. (J) Tumor growth curves depicting the growth patterns of nude mice across various treatment groups. (K) Body weight fluctuations observed in nude mice subjected to different treatment protocols. (L) Comparison of tumor weights in nude mice receiving different treatments. Quantitative flow cytometry analysis results are presented below. Data are presented as mean ± standard error of the mean (SEM). Statistical significance is denoted as *p < 0.05, **p < 0.01, ***p < 0.001.

PD‐1 on tumor‐infiltrating lymphocytes binds to PD‐L1 on the surface of cancer cells, where it transmits negative regulatory signals, preventing T cells from recognizing tumor cells to exert antitumor activity and resulting in immune escape.^38^ To determine the ability of TF to enhance the cytotoxic effect of T cells on tumor cells, we cocultured TF‐treated RKO and HT29 cells with Jurkat cells overexpressing PD‐1. As shown in Figure 2A,B, TF reactivated T cells and significantly reduced RKO and HT29 cell survival when compared with control cells. In summary, TF can relieve the immune suppression of tumor cells to T cells by reducing the level of PD‐L1 on the surface of tumor cells and subsequently enhancing tumor immunity.

### 2.3 TF suppresses tumor growth in vivo by activating tumor‐infiltrating T cells

To further substantiate the tumor‐suppressive effects of TF in immunocompetent C57 mice, we performed daily intraperitoneal injections of TF at doses of 12.5 or 25 mg/kg for a period of 12 days (Figure 2C). Tumors in the PBS group grew rapidly, while tumor growth was effectively inhibited after treatment with 12.5 or 25 mg/kg TF. Moreover, both the 12.5 and 25 mg/kg treatments showed significant tumor suppression efficiency, with suppression rates of 59.28 and 20.81%, respectively. In addition, there was little change in the weight of the mice throughout the entire treatment period (Figure 2E–H). Subsequently, we further confirmed the inhibitory effects of TF on tumor growth in immunodeficient mice by administering PBS or TF (25 mg/kg) via daily intraperitoneal injections for 14 days (Figure 2D). Interestingly, the suppressive effect of TF on the growth of MC38 subcutaneous tumors was no longer detected in nude mice lacking T cells (Figure 2I–L), indicating that the therapeutic effectiveness of TF against tumors primarily stems from the activation of T cell immunity induced by TF. Furthermore, H&E staining was used for histological examination of principal organs in mice, and no significant harmful effects were observed with TF at therapeutic doses (Figure S3).

We analyzed tumor‐infiltrating immune cells to observe the effect of TF on immune cells in the tumor immune microenvironment. We found that the populations of activated regulatory T cells (Tregs) (CD4^+^CD25^+^FoxP3^+^) and MDSCs (CD11b^+^Gr‐1^+^) within the TILs were considerably lower in the mice treated with TF than in the control group. Granzyme B is a serine protease released from cytoplasmic granules in cytotoxic T cells and NK cells and is associated with T cell‐mediated cytotoxicity. The elevated levels of GzmB further demonstrated that TF enhances cytotoxic T lymphocyte activity (Figure S4A,B). In addition, immunohistochemical analysis of tumors revealed that TF concentration‐dependently increased the levels of CD3 (a marker of T cells), CD4 (a Treg), CD8 (a cytotoxic T cell), and C‐caspase‐3 (a mitotic caspase‐3) in tumors, whereas the levels of FoxP3 (an immunosuppressive molecule), Ki‐67 (a marker of proliferation), and PD‐L1 were markedly reduced (Figure S4C,D). Collectively, our findings indicate that TF may relieve the immunosuppressive effect on T cells by reducing the levels of PD‐L1 in cancer cells, thereby increasing the infiltration of T cells into the tumor tissue.

### 2.4 TF degrades PD‐L1 via the ubiquitin–proteasome pathway

**FIGURE 3:**
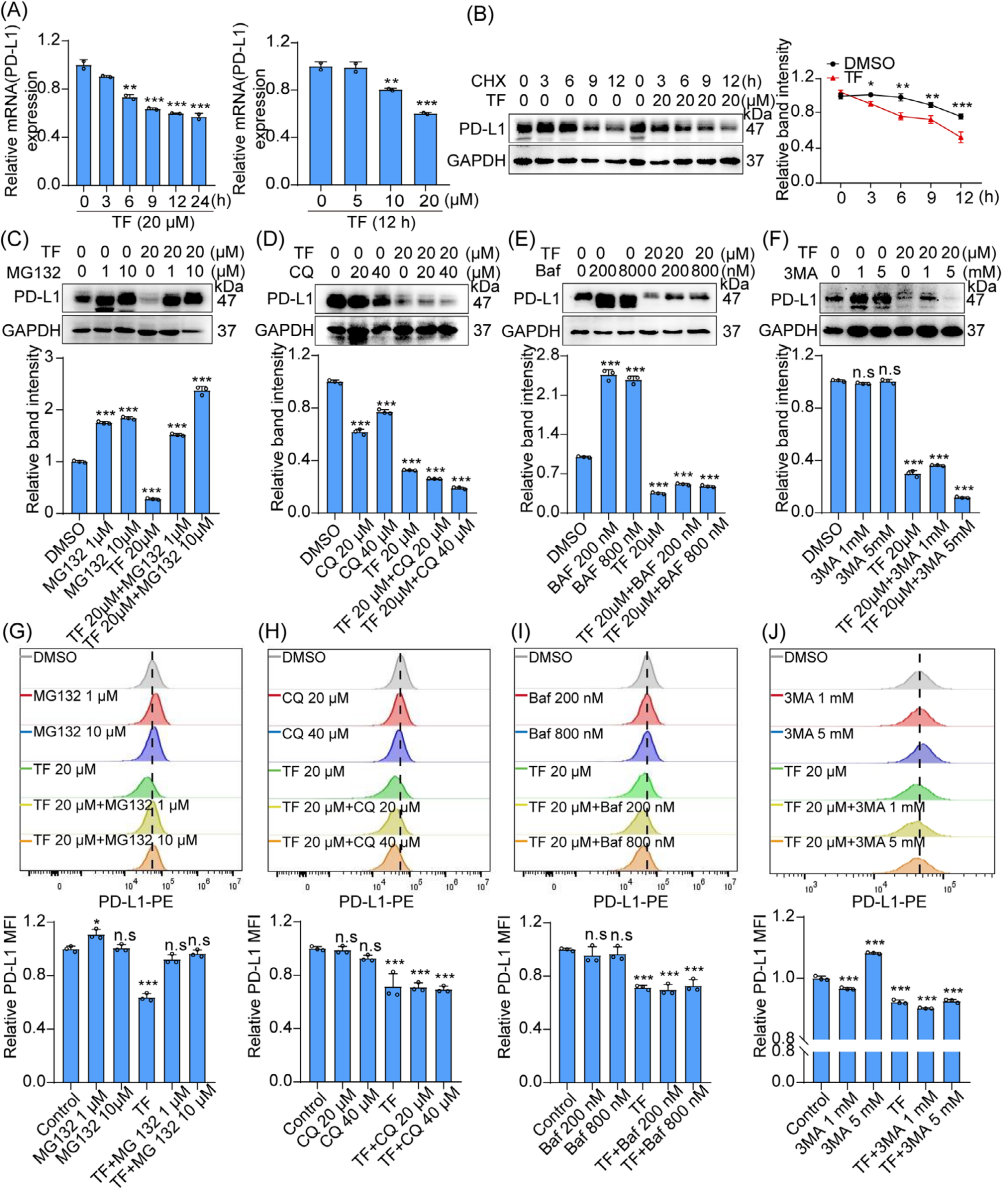
TF induces PD‐L1 degradation via the ubiquitin–proteasome pathway. (A) Quantitative RT‐PCR assessed PD‐L1 mRNA levels in RKO cells treated with various concentrations of TF for different durations. (B) Immunoblotting detected PD‐L1 expression in RKO cells treated with either DMSO or 20 µM TF alongside cycloheximide (CHX) (50 mg/mL). Quantitative analysis results are provided. (C–F) Immunoblotting revealed PD‐L1 expression in RKO cells treated with TF in combination with the proteasome inhibitor MG132 (C), chloroquine (CQ) (D), the lysosome inhibitor bafilomycin (Baf) (E), or the autophagy inhibitor 3‐methyladenine (3‐MA) (F). Quantitative analysis of the immunoblotting results is presented below. (G–J) Flow cytometry assessed PD‐L1 expression on the membrane surface of RKO cells treated with TF in combination with MG132 (G), CQ (H), Baf (I), or 3‐MA (J). Quantitative flow cytometry analysis results are displayed below. Data are presented as mean ± standard error of the mean (SEM). Statistical significance is denoted as *p < 0.05, **p < 0.01, ***p < 0.001.

To investigate how TF regulates the degradation of PD‐L1 at the molecular level, we first analyzed the levels of PD‐L1 at the transcriptional and translational levels in RKO cells treated with TF. Our findings showed that TF treatment resulted in a time‐ and dose‐dependent decrease in the mRNA level of PD‐L1, as demonstrated by real‐time PCR analysis (Figure 3A). Additionally, real‐time PCR results showed that the expression of the PD‐L1 transcription factors IRF1, STAT1, and NRF2 decreased after TF treatment, suggesting that TF may downregulate PD‐L1 expression at the transcriptional level (Figure S5A,B). Interestingly, when PD‐L1 was combined with cycloheximide (CHX), PD‐L1 had a shorter half‐life in the TF‐treated groups than in the untreated groups (Figure 3B), indicating that TF might also modulate PD‐L1 via posttranslational regulatory processes. After 3 h of incubation with TF, CRC cells exhibited a notable decrease in PD‐L1 expression, while TF had little effect on the expression level of PD‐L1 mRNA. Therefore, we hypothesized that TF degrades PD‐L1 mainly at the posttranslational level. In subsequent studies, we will focus on the posttranslational modification mechanisms through which TF participates in the degradation of PD‐L1 in tumors.

The degradation pathways of PD‐L1 mainly include the proteasomal degradation pathway and lysosomal degradation pathway.^17^, ^20^, ^21^, ^39^ To determine which pathway TF regulates PD‐L1 degradation, we cotreated RKO cells with TF and different inhibitors, such as lysosomal inhibitor chloroquine/bafilomycin (CQ/Baf), the autophagy inhibitor 3‐methyladenine (3‐MA) and proteasome inhibitor MG132, and IB experiments showed that the proteasome inhibitor MG132 reversed the degradation of PD‐L1 by TF but not by CQ, Baf, or 3‐MA (Figure 3C–F). Consistent with the IB results, the immunofluorescence results also showed that the proteasome inhibitor MG132 reversed the effect of TF on PD‐L1 degradation (Figure S5C,D). In addition, flow cytometric analysis demonstrated that the proteasome inhibitor MG132 reversed the regulatory impact of TF on the degradation of PD‐L1 (Figure 3G–J). TF had almost no effect on lysosomal function, which further confirmed that the ubiquitin–proteasome pathway is involved in the degradation of PD‐L1 by TF (Figure S5E,F). Taken together, these results consistently suggested that the ubiquitin–proteasome pathway plays a role in the TF‐mediated degradation of PD‐L1.

### 2.5 TF degrades PD‐L1 by targeting and stabilizing HRD1

**FIGURE 4:**
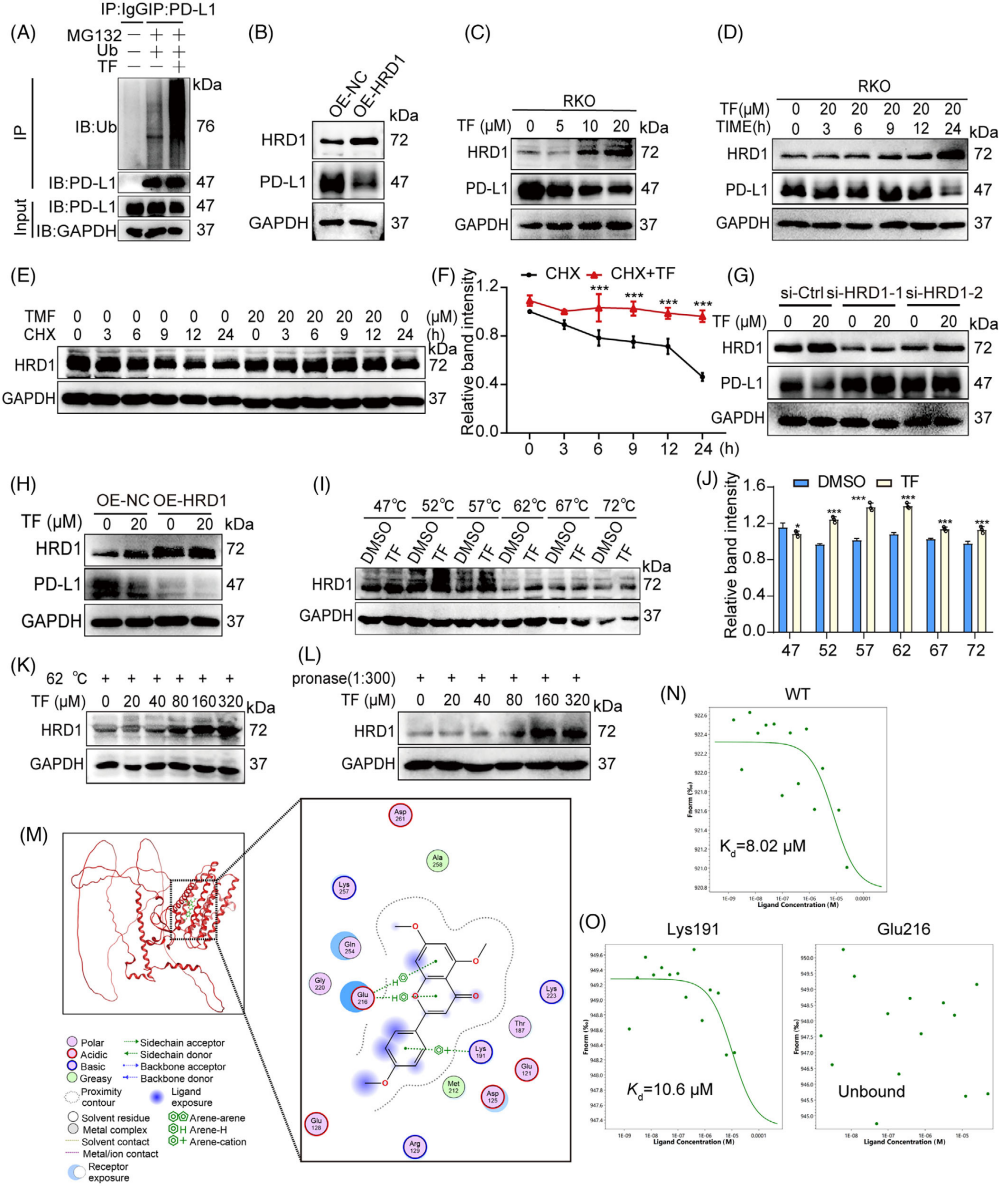
TF directly binds and promotes HRD1 activity. (A) Ub was overexpressed in RKO cells to detect the TF‐induced ubiquitination of PD‐L1. Immunoprecipitation of ubiquitinated PD‐L1 protein was performed using Flag bead pellets and immunoblotting with an Ub antibody. (B) HRD1 was overexpressed in RKO cells, and the effect of HRD1 on PD‐L1 was detected by immunoblotting. (C and D) RKO cells were treated with various concentrations of TF for 24 h or a fixed dose of 20 µM for the indicated durations. Immunoblotting was used to measure the total HRD1 protein levels. (E) Immunoblot analysis was conducted to evaluate the levels of HRD1 in RKO cells treated with either DMSO or 20 µM TF along with cycloheximide (CHX) (50 mg/mL). (F) The results of the quantitative analysis of IB for (E). (G) PD‐L1 protein expression in TF‐treated RKO cells was detected by immunoblotting analysis in the presence of siRNA targeting HRD1 or a negative control (siRNA‐Ctrl). (H) PD‐L1 protein expression in TF‐treated RKO cells was detected by immunoblotting analysis in the presence of HRD1 overexpression in RKO cells or negative controls (OE‐NC). (I) The CETSA method was used to determine the thermal stability of HRD1 when interacting with TF at temperatures ranging from 47 to 72°C. (J) The results of the quantitative analysis of IB for (I). (K) Stability of HRD1 to different concentrations of TF at 62°C. (L) Stability of HRD1 to different concentrations of TF when the ratio of pronase to protein was 1:300. (M) Molecular docking of TF to HRD1. (N) Binding of wild‐type HRD1 to TF. (O) Binding of TF to HRD1 with mutations at sites Lys191 or Glu216. Data are presented as mean ± standard error of the mean (SEM). Statistical significance is denoted as *p < 0.05, **p < 0.01, ***p < 0.001.

Previous reports have confirmed that the modulation of PD‐L1 through the ubiquitin/proteasome pathway is mainly mediated by deubiquitinating enzymes or E3 ubiquitin ligases.^14^ We first performed IP analysis and showed that treatment with TF increased the ubiquitination level of PD‐L1 in both RKO and MC38 cells, which further confirmed that TF enhanced the degradation of PD‐L1 via the ubiquitin–proteasome pathway (Figures 4A and S5G). Subsequently, we screened E3 ubiquitin ligases associated with PD‐L1 by small interfering RNA and found that none of the E3 ubiquitin ligases other than HRD1 mediated the degradation of PD‐L1 by TF (Figure S6). Furthermore, PD‐L1 expression was downregulated when HRD1 was overexpressed (Figures 4B and S7A). Since HRD1 is a ubiquitin ligase for PD‐L1, we next investigated whether TF affects HRD1. As shown in Figures 4C,D and S7B,C, TF increased HRD1 expression in a dose‐ and time‐dependent manner. Additionally, CHX experiments revealed that the breakdown of HRD1 in TF‐treated cells was slower than that in control cells (Figure 4E,F). We also detected the influence of TF on HRD1 mRNA levels and found no significant change in HRD1 transcription after the administration of TF (Figure S7D). Moreover, when HRD1 was silenced by siRNA, PD‐L1 degradation was reversed, and overexpression of HRD1 decreased PD‐L1 expression (Figures 4G,H and S7E,F). Collectively, this evidence indicated that HRD1 is involved in the TF‐induced destabilization of PD‐L1.

We hypothesized that TF might bind directly to HRD1, and subsequently, we performed cellular thermal shift assay (CETSA) experiments to detect direct drug‐protein interactions. As shown in Figure 4I,J, TF significantly increased the accumulation of HRD1, and the stability of HRD1 at 62°C increased with increasing TF concentration (Figures 4K and S7G). Furthermore, we also observed a concentration‐dependent accumulation of HRD1 with increasing TF concentration at a streptavidin:protein lysate ratio of 1:300 (Figures 4L and S7H).

To identify the specific amino acid residues within HRD1 that interact with TF, we conducted docking simulations using Molecular Operating Environment (MOE) software (Figure 4M). Our simulations indicated potential interactions between TF and residues such as Glu216 and Lys191. To further elucidate the binding sites of TF and HRD1, we generated GFP‐tagged wild‐type, Glu216, and Lys191 mutant HRD1 plasmids and subsequently analyzed direct binding by microscale thermophoresis (MST). The *K*_d_ value for TF binding to the wild‐type HRD1 protein was estimated to be 8.02 µM, indicating a strong binding affinity (Figure 4N). Mutation of the Lys191 site in the HRD1 protein did not affect its binding to TF (Figure 4O). However, when the Glu216 site was mutated, the binding interaction between TF and HRD1 disappeared (Figure 4O). These results suggest that TF binds to HRD1 via the Glu216 site.

**FIGURE 5:**
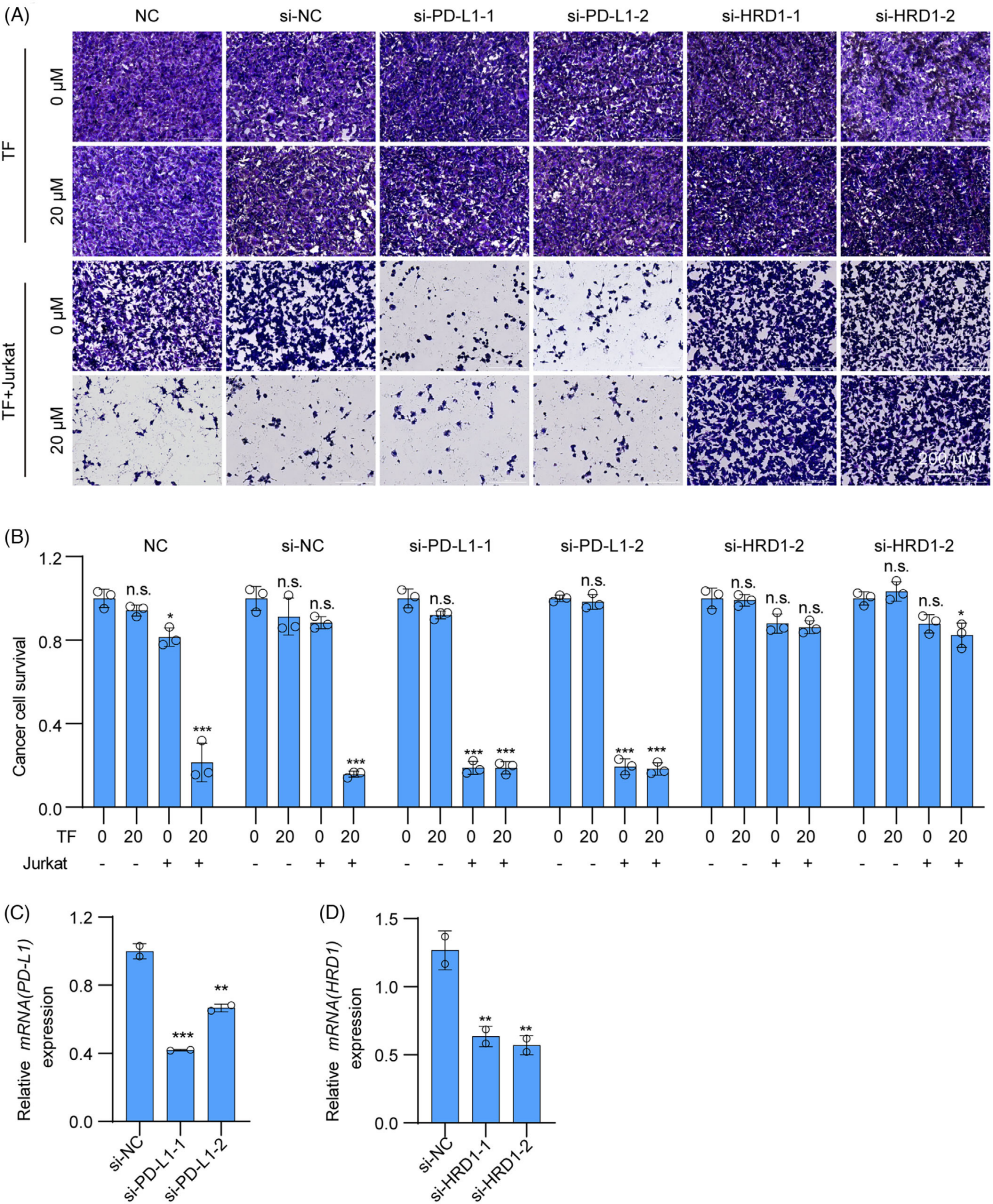
TF promotes T cell killing in vitro by targeting HRD1 ubiquitination to degrade PD‐L1. (A) Jurkat cells cocultured with RKO cells were treated with TF for 24 h. Surviving tumor cells were observed via crystal violet staining. (B) Quantitative plot corresponding to (A). (C and D) siRNA knockout inefficiency. Data are presented as mean ± standard error of the mean (SEM). Statistical significance is denoted as *p < 0.05, **p < 0.01, ***p < 0.001.

To demonstrate that TF exerts a tumor immune effect by targeting HRD1 to degrade PD‐L1 in tumors, we first interfered with PD‐L1 in RKO cells and found that Jurkat cells had almost the same ability to kill RKO cells when treated with TF alone, TF+siPD‐L1, or siPD‐L1 alone, which further confirmed that TF enhances T cell killing by reducing PD‐L1 in tumors. Next, we interfered with HRD1 in RKO cells and found that neither TF plus siHRD1 nor siHRD1 alone could enhance the cytotoxic effect of T cells on RKO cells, suggesting that TF mediates T cell killing through HRD1 (Figure 5). In summary, TF promotes T cell‐mediated antitumor immunity via PD‐L1 degradation in cancer cells by targeting HRD1.

### 2.6 TF is comparable to either anti‐CTLA‐4 alone or anti‐PD‐1 alone for antitumor immunity

**FIGURE 6:**
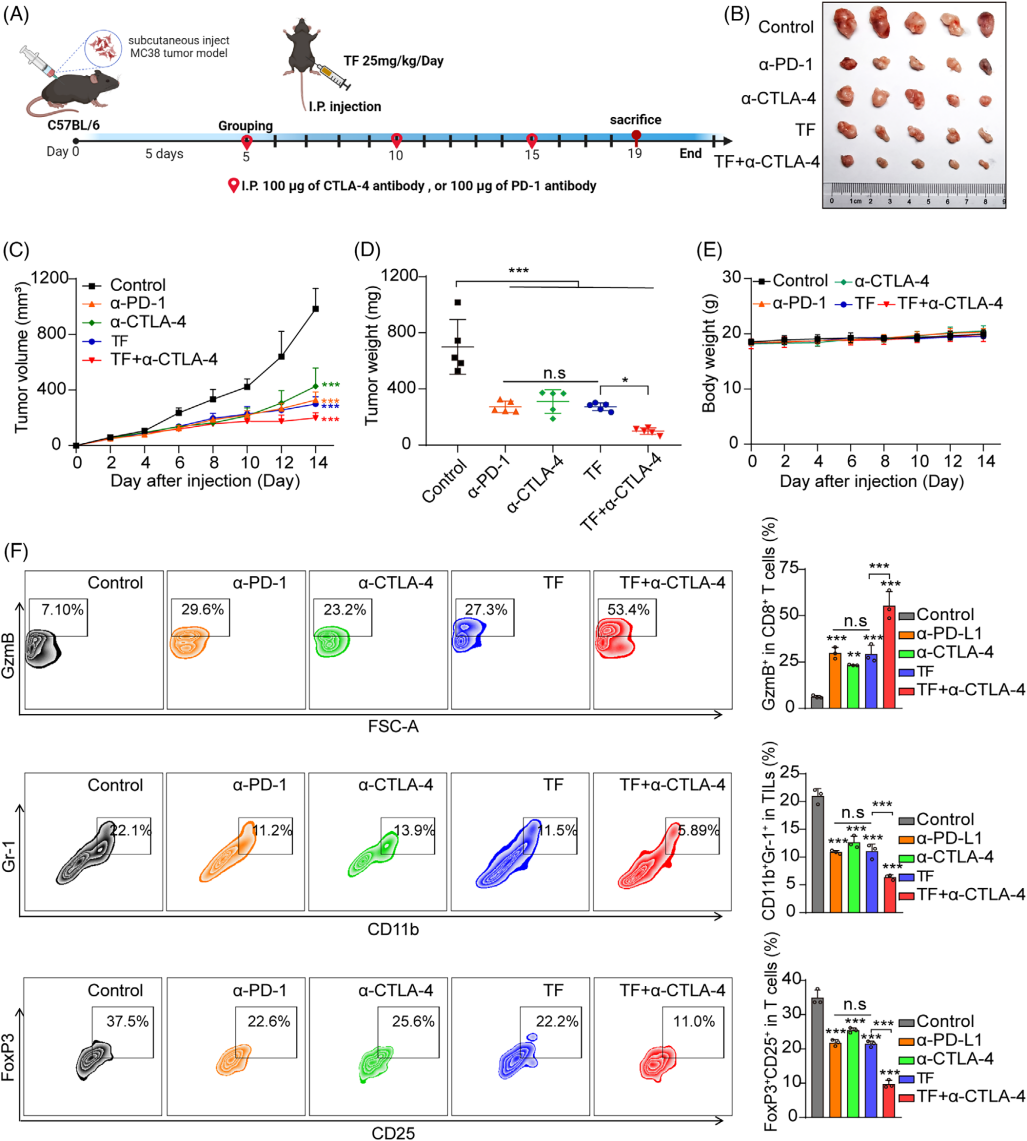
TF is as effective as anti‐PD‐1 or anti‐CTLA‐4 therapy alone for antitumor immunity. (A) Experimental procedures for animal experiments in C57BL/6J (female) mice (i.p. intraperitoneal injection). Ex vivo observation of the tumors from the treated mice (PBS, anti‐PD‐1, anti‐CTLA4, TF (25 mg/kg), or TF (25 mg/kg) combined with anti‐CTLA4) was performed. (B) Visual depiction of typical solid tumors observed in C57BL/6 mice across various treatment cohorts. (C) Growth trajectories of tumors in C57BL/6J mice subjected to different treatment regimens. (D) Comparative analysis of tumor masses among C57BL/6J mice exposed to distinct treatment modalities. (E) Tracking of body weight changes in C57BL/6J mice across different treatment conditions. (F) Representative flow cytometry maps of the CD4^+^CD25^+^FoxP3^+^ and CD11b^+^ Gr‐1^+^ populations and GzmB levels in CD3^+^CD8^+^ TILs from MC38 tumors treated with different therapeutic approaches. Data are presented as mean ± standard error of the mean (SEM). Statistical significance is denoted as *p < 0.05, **p < 0.01, ***p < 0.001.

PD‐1 and CTLA‐4 are commonly used checkpoint inhibitors in clinical practice, and TF also exerts antitumor effects through immune checkpoints. Therefore, it is necessary to compare the immune antitumor effects of TF and anti‐PD‐1 alone or anti‐CTLA‐4 alone or TF plus anti‐CTLA‐4. As shown in Figure 6A–E, we subcutaneously loaded female C57BL/6J mice with MC38 cells to induce tumor loading and divided the mice into five groups: PBS, TF, anti‐CTLA‐4, anti‐PD‐1, and TF plus anti‐CTLA‐4. According to the findings from the experiments, we found that either TF alone or antibody treatment alone significantly inhibited the tumor growth rate. When comparing whether TF treatment was superior to anti‐CTLA‐4 or anti‐PD‐1 treatment, the results showed that although TF treatment alone and anti‐PD‐1 treatment alone were slightly better than anti‐CTLA‐4 treatment alone, there was no statistically significant difference among the three groups. Since anti‐CTLA‐4 and anti‐PD‐1 are two different immune checkpoints and the combination of both has already shown significant therapeutic effects in terms of overall survival and response rates in cancer patients,^40^ we also explored whether TF combined with CTLA‐4 antibody therapy has synergistic antitumor effects. Our subsequent investigations focused on determining whether TF combined with CTLA‐4 antibody treatment could produce synergistic antitumor effects. We observed a more significant antitumor effect of TF combined with CTLA‐4 than of TF alone, and the body weights of the mice were not affected (Figure 6B–E). As shown in Figure 6F, the combination treatment group had the lowest proportions of Tregs (CD4^+^CD25^+^FoxP3^+^) and MDSCs (CD11b^+^Gr‐1^+^) compared with the other groups. Furthermore, the combination treatment group also exhibited the highest levels of GzmB.

Immunohistochemical analysis revealed that the PD‐L1 level in the tumor tissue significantly decreased after TF treatment, while the HRD1 level significantly increased (Figure S8A,B). In addition, TF is comparable to CTLA‐4 or PD‐1 antibodies used alone for increasing T cell infiltration and promoting tumor cell apoptosis, while the combination of TF and CTLA‐4 antibodies is more effective (Figure S8C,D). To further investigate the changes in other immune cells in the tumor microenvironment after TF administration, NK cells, macrophages, and dendritic cells (DCs) were detected by immunohistochemistry. By analyzing DCs, we found that the proportion of CD11c^+^ cells increased significantly, indicating an enhanced antigen presentation capacity. In addition, the percentage of M1 macrophages, characterized by CD86 and F480 positivity, increased considerably following TF treatment. Conversely, there was a notable reduction in the percentage of M2 macrophages, which were identified by CD206 positivity, indicating that TF reduced tumor PD‐L1 to relieve T cell immune suppression while also polarizing macrophages toward the M1 phenotype. The increased number of tumor‐infiltrating NK (CD56^+^) cells also confirmed that TF treatment further enhanced tumor immunity (Figure S9A,B). Similarly, H&E staining revealed no significant toxic effects on the major organs of the mice after combination treatment (Figure S10). In summary, TF is a promising natural small‐molecule compound for tumor immunotherapy, and when combined with an anti‐CTLA‐4 antibody, it shows a stronger ability to inhibit CRC cell growth, which in turn stimulates a more vigorous antitumor immune reaction.

### 2.7 HRD1 and PD‐L1 expression in human cancerous tissues

**FIGURE 7:**
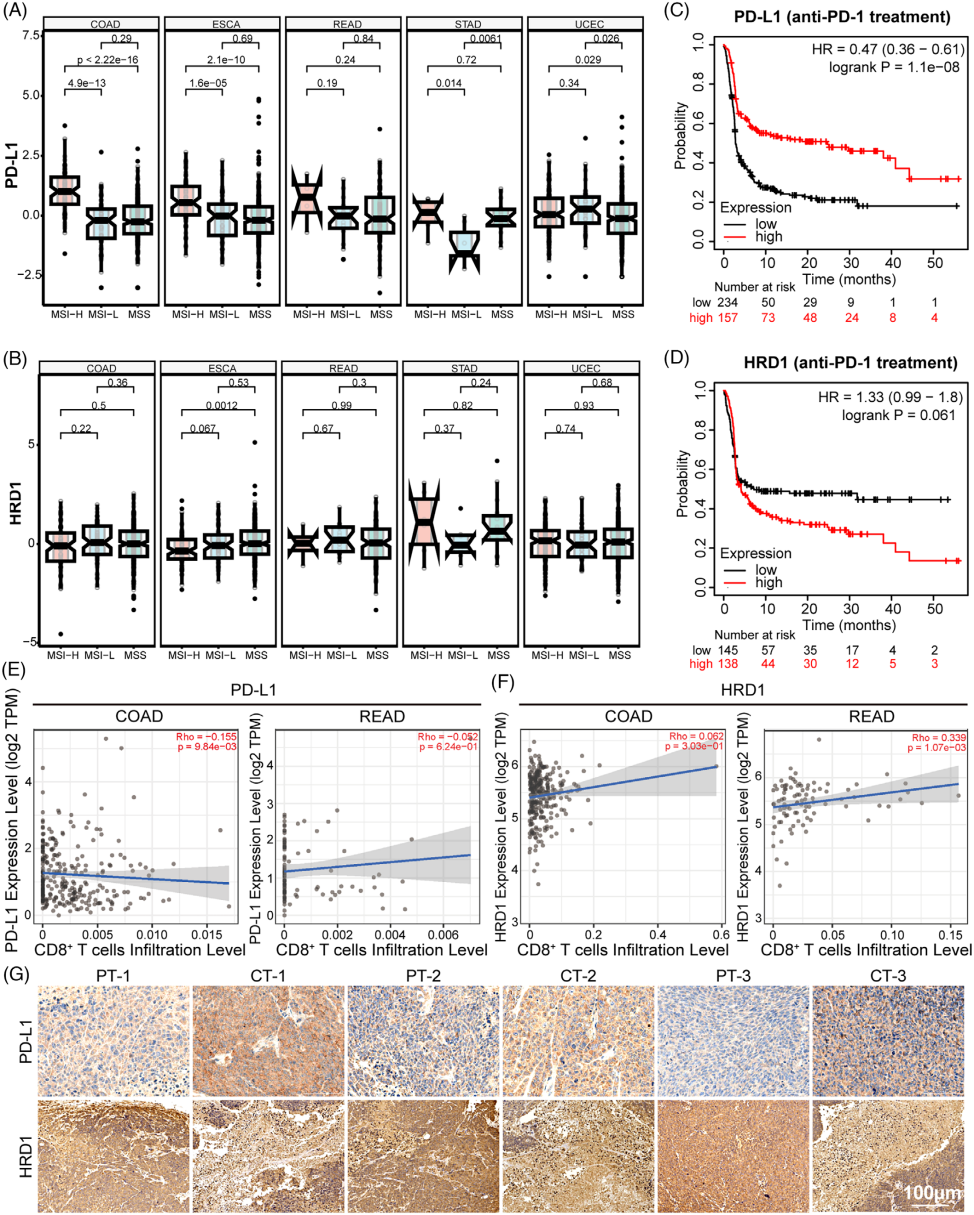
The association between PD‐L1 and HRD1 expression in CRC tissues. (A) PD‐L1 expression in patients with different molecular subtypes (microsatellite instability). (B) Evaluation of HRD1 expression across various molecular isoforms (microsatellite instability). (C) Comparison of survival outcomes among colorectal cancer (CRC) patients stratified based on PD‐L1 expression using two‐sided log rank analysis. (D) Assessment of survival rates in CRC patients categorized by HRD1 expression utilizing two‐sided log rank analysis. (E) Scatter plots depicting the association between PD‐L1 expression and infiltrating CD8+ T cells in COAD or READ patients as analyzed by TIMER 2.0. (F) Visualization of the correlation between HRD1 expression and infiltrating CD8+ T cells in COAD or READ patients using scatter plots generated by TIMER 2.0. (G) Illustrative IHC images demonstrating PD‐L1 and HRD1 staining patterns in paracancerous tissues (PT) and cancer tissues (CT) from individuals with CRC.

To investigate the relationship between PD‐L1 and HRD1 levels in human cancerous tissues, we analyzed their expression in different microsatellite molecular subtypes of various tumors using the Sparkle database. In CRC patients, the high‐frequency microsatellite unstable molecular type was associated with a better immune response. As shown in Figure 7A,B, in the MSI‐H molecular subtype, colorectal adenocarcinoma (COAD), esophageal carcinoma, and rectal adenocarcinoma (READ) had high levels of PD‐L1 and were inversely related to HRD1 expression. However, this pattern was not observed in uterine stomach adenocarcinoma or corpus endometrial carcinoma. Subsequently, an examination of The Cancer Genome Atlas database revealed a negative correlation between the efficacy of anti‐PD‐1 therapy and the expression levels of PD‐L1 and HRD1 in individuals with colon cancer. Individuals with elevated PD‐L1 levels and reduced HRD1 levels exhibited prolonged survival, as depicted in Figure 7C,D. TIMER 2.0 was used to evaluate the interplay between immune cell infiltration and the expression levels of PD‐L1 and HRD1 in patients with COAD and READ. As illustrated in Figure 7E,F, there was an adverse association between CD8^+^ T cell infiltration and PD‐L1 expression, whereas an affirmative correlation was observed between CD8^+^ T‐cell infiltration and HRD1 expression. These outcomes imply that tumors with elevated PD‐L1 and diminished HRD1 may suppress T cell activation within the tumor's immunological microenvironment.^41^, ^42^, ^43^ Furthermore, a comparative assessment was performed on the expression levels of PD‐L1 and HRD1 in adjacent and tumor tissues of patients diagnosed with colon cancer. As illustrated in Figure 7G, HRD1 expression was notably higher in the surrounding tissues compared with the cancerous tissues, while PD‐L1 expression exhibited lower levels in the adjacent tissues compared with the tumor tissues. Taken together, our findings suggest that HRD1 may exert an inhibitory influence on PD‐L1 expression within tumor tissues.

## 3 DISCUSSION

Compared with the use of anti‐PD‐1/PD‐L1 monoclonal antibodies, the use of small molecules to downregulate PD‐L1 expression is also an effective means of enhancing immunotherapy efficacy. Natural small molecule compounds have great potential for development as PD‐1/PD‐L1 inhibitors due to their low cost, high oral utilization, and abundance. Many natural small molecule compounds can effectively mediate PD‐L1 expression and exert antitumor immune effects. For example, berberine and curcumin target the deubiquitinating enzyme CSN5 to mediate the expression of PD‐L1 and participate in tumor immunity.^26^, ^41^ Tubeimoside‐1 plays an antitumor immune role through the lysosomal pathway to mediate PD‐L1 expression.^23^ Our previous findings showed that the natural marine product benzosceptrin C can induce lysosomal degradation of PD‐L1 by inhibiting DHHC3 enzymatic activity.^42^ Recent research has indicated that TF exerts an antineoplastic effect by promoting apoptosis in tumor cells. However, our results showed that TF is capable of markedly decreasing PD‐L1 expression on the surface of cancer cells, which subsequently disrupts its interaction with PD‐1 on T cells and reinvigorates the antitumor immune response, suggesting its broad potential in tumor immunotherapy. In addition, CCK‐8 and EdU experiments showed that TF at effective concentrations had no significant toxic effects on cells.

Our study showed that TF induced T cell immunity by mediating PD‐L1 degradation. First, we found that TF could enhance T cell killing of tumor cells in in vitro T cell killing assays. In addition, the antitumor effect of TF disappeared in immunodeficient mice; however, TF had an antitumor effect on C57BL/6J mice. Further studies revealed that TF suppressed the expression of Tregs (CD4^+^CD25^+^FoxP3^+^) and MDSCs (CD11b^+^Gr‐1^+^) by promoting the production of GzmB, thereby inducing T‐cell activation and exerting antitumor immune effects more effectively. Therefore, our study identified TF as a potential tumor immunotherapy drug.

Numerous investigations have demonstrated that PD‐L1 undergoes degradation via the ubiquitin/proteasome pathway and that E3 ubiquitin ligases can be involved in this pathway for relevant regulation. Therefore, we initially hypothesized that targeting E3 ubiquitin ligases might be a new target for regulating PD‐L1. HRD1 is an ER‐related E3 ubiquitin ligase; interestingly, HRD1 can participate in the regulation of ubiquitin/proteasome pathway‐mediated degradation of PD‐L1, thereby exerting antitumor immune effects. In this study, the potential targets of PD‐L1 in TF regulation were explored via gene interference technology. An important finding is that TF can specifically bind and stabilize the E3 ubiquitin ligase HRD1, thereby further promoting the ubiquitination of PD‐L1 and promoting PD‐L1 degradation. Moreover, PD‐L1 expression was more stable when PD‐L1 was knocked down, whereas TF stabilized and upregulated HRD1 protein decreased PD‐L1 expression, suggesting that targeting HRD1 could have a significant therapeutic effect on tumors. To further investigate whether TF can directly target HRD1 to mediate PD‐L1 ubiquitination, we used a CETSA to verify that TF can directly bind to and stabilize HRD1, leading to PD‐L1 degradation. This provides a potential regulatory target for TF in PD‐L1 regulation. Additionally, using molecular docking and MST, we predicted and verified the binding site of TF with HRD1 at the Glu216 site, providing compelling evidence for the possible application of TF as an immunotherapeutic small molecule. Additionally, our research indicated that TF influences the transcriptional level of PD‐L1, and subsequent investigations showed that TF can impact the expression of transcription factors associated with PD‐L1. Thus, TF is likely capable of regulating PD‐L1 expression through both transcriptional and posttranslational pathways, suggesting that TF may have a more potent capacity to control PD‐L1 reduction than other small molecules. Nevertheless, the precise mechanisms by which TF alters PD‐L1 transcript levels remain unclear and warrant additional research and in‐depth exploration in future studies.

The use of ICB in tumor immunotherapy has been found to enhance antitumor T‐cell responses through anti‐PD‐1, anti‐CTLA‐4, or anti‐PD‐L1 therapy. When comparing the efficacy of TF with that of anti‐PD‐1 or anti‐CTLA‐4, the findings indicated that TF alone was as effective as anti‐CTLA‐4 or anti‐PD‐L1 treatment alone, suggesting that TF has significant clinical potential. Since TF promotes the degradation of PD‐L1 protein in tumor cells, which then blocks the PD‐L1/PD‐1 pathway. Therefore, we hypothesized that TF combined with anti‐CTLA‐4 binding could enhance the antitumor T‐cell response and produce synergistic antitumor effects. Our research using an MC38 mouse subcutaneous tumor model indicated that the synergistic use of TF and anti‐CTLA‐4 led to a pronounced increase in CD8^+^ T‐cell infiltration and a suppression of tumor growth compared with the effects of TF, anti‐PD‐1, or anti‐CTLA‐4 alone. Furthermore, the combination of TF and an anti‐CTLA‐4 antibody did not lead to any significant toxic effects. Our findings suggest that TF combined with anti‐CTLA‐4 treatment has strong potential for enhancing the antitumor effects of TF in the clinic.

Previous studies have shown that the oral absorption of TF is slow when it is administered as an aqueous solution, whereas the oral bioavailability of TF is significantly increased when it is administered as a solution formulated with 2‐hydroxypropyl‐β‐cyclodextrin.^43^ Therefore, the use of lipid solutions may optimize the efficacy of TF in subsequent formulation or pharmacological studies. The elimination capacity of TF is saturable, and TF elimination is autoinducing, suggesting that the pharmacokinetic profiles of drugs sharing the same elimination pathway as TF may be altered after prolonged exposure to TF.^44^ Future studies should focus on elucidating the elimination pathway of TF to maximize its pharmacological effects.

There are certain limitations to this study. First, the study only examined the effectiveness of TF in treating CRC, and further research is needed to investigate its efficacy in treating other types of cancer. Second, TFs have a particular impact on the transcription level of PD‐L1, and further research on the transcription factors that are influenced by TF is needed.

In conclusion, our findings suggest that TF can exert an antitumor effect by targeting and stabilizing HRD1 and subsequently degrading PD‐L1 through the ubiquitin–proteasome pathway. Furthermore, in vivo studies indicated that TF can reduce the expression of tumor PD‐L1, relieve the immunosuppression of T cells, enhance tumor‐infiltrating T‐cell viability, and subsequently produce antitumor effects. These results lay the foundation for the potential use of TF as a small molecule inhibitor to disrupt PD‐L1‐mediated immune evasion.

## 4 MATERIALS AND METHODS

### 4.1 Antibodies and reagents

TF was purchased from Dingrui Chemical (Shanghai). MG132, Baf, CQ, CHX, and 3MA were obtained from MedChemExpress (Monmouth Junction, NJ, USA). The reverse transcription kit was purchased from Yeasen (Cat #11119; Shanghai, China). Recombinant human IFN‐γ was purchased from PeproTech (NJ, USA). The antibodies used are listed in Table S1.

### 4.2 Cell viability and proliferation assay

Cell viability and proliferation assays were conducted following the protocols outlined in the CCK‐8 and EdU kit, respectively. Cell viability was determined by calculating IC50 values using the logarithmic method based on CCK‐8 results, while proliferation was evaluated by capturing images using a high‐content imaging system. The kits utilized in these assays were procured from Beyotime (Haimen, China).

### 4.3 Flow cytometry and immunofluorescence

Flow cytometry was used to examine PD‐L1 expression on cell membranes. Following cell treatment, the cells were incubated with anti‐PD‐L1 fluorescent antibody at 4°C for 30 min, washed with PBS, and resuspended before analysis on a flow cytometer. Immunofluorescence staining was also utilized to evaluate PD‐L1 expression on cell membranes. The cells were incubated overnight at 4°C with anti‐PD‐L1 antibody, followed by incubation with the appropriate fluorescent secondary antibody in the dark. DAPI staining was applied to visualize the nuclear structure of the cells. Images were captured using a Cytation 5 (BioTek, USA).

### 4.4 T‐cell culture and T‐cell–mediated tumor cell killing assay

Tumor cells treated with TF were cocultured with Jurkat cells transfected with PD‐1. Before coculture, Jurkat cells were activated with 1 mg/mL phytohemagglutinin and 50 ng/mL phorbol 12‐myristate 13‐acetate. The ratio of tumor cells to Jurkat cells was 1:6. Upon completion of the experiment, imaging was conducted using crystal violet staining with a Cytation 5 imaging system (BioTek).

### 4.5 Cellular thermal shift assay

Adequate RKO cells were treated with NP40 to achieve lysis, after which the resulting protein lysate was equally divided into two portions. TF at a concentration of 200 µM was introduced to one portion, while the other portion received an equivalent volume of DMSO. These mixtures were then allowed to stand for 20 min at ambient temperature. Subsequently, the samples were aliquoted into 70 µL per tube, subjected to a temperature gradient starting at 5°C, and heated at various temperatures for 3 min. After this, the samples were chilled on ice, followed by centrifugation to pellet any precipitated material. The supernatant was then subjected to SDS‐PAGE for protein separation and further analyzed via Western blotting using an antibody specific for HRD1.

### 4.6 Microscale thermophoresis

GFP target protein plasmids were constructed, and the GFP HRD1 plasmid and mutant plasmids were expressed in 293T cells. After 48 h, the cells were lysed, and cell lysates were obtained. Assays were performed using a MonolithTM NT.115 MST device (NanoTemper, Germany).

### 4.7 Molecular docking of TF to HRD1

The amino acid sequence of HRD1 was acquired from UniProt (code: Q86TM6), and a 3D molecular docking model of compound TF with HRD1 was generated using Alpha‐Fold and MOE. Following energy minimization, potential binding sites for small molecules on HRD1 were predicted using the SiteFinder module in MOE, and molecular docking calculations were conducted using the DOCK module in MOE. Subsequent to the molecular docking calculations, the most favorable docking results were selected for further analysis of the binding modes of compounds TF and HRD1.

### 4.8 Tumor‐infiltrating lymphocyte isolation and T‐cell profiling

To assess the immunoindex of animal tumor samples, MC38 tumors from each group were collected, cut into paste, and then lysed and digested with collagenase 4 (1 mg/mL; Yeasen) and DNase 1 (0.1 mg/mL; Yeasen) for 2 h at 37°C. The lysed cytosol was filtered through a 70 µM membrane, washed with PBS, stained with antibodies against CD3, CD8, CD4, CD25, Gr‐1, CD11b, and GzmB, washed with 1×TF wash buffer, and centrifuged at 1000 rpm for 3 min, after which the supernatant was discarded. The nuclei were then processed for 30 min, and the nucleation reagent was prepared by diluting solution B (TF Fixlperm Buffer 4×) to 1× with solution A (TF Diluent Buffer). At the end of nucleation, 1 µL of TF wash buffer was added for washing, and the supernatant was removed by centrifugation at 1000 rpm for 3 min. Subsequently, FoxP3 antibody was added for staining for 30 min, 1× TF wash buffer was added for washing, the mixture was centrifuged at 400 g for 3 min, the cells were resuspended in PBS containing 2% serum, and then all the samples were analyzed by flow cytometry (Beckman‐Coulter, USA). The data were analyzed using FlowJo software.

Please see the Supporting Information for the details of cell, Western blotting and immunoprecipitation, RNA extraction and real‐time PCR analysis, transfection, and mouse methods.

### 4.9 Statistical analysis

Statistical analyses were conducted using GraphPad Prism 8.0.1 or SPSS 23 software. Data are presented as mean ± standard error of mean (SEM). Independent samples t‐tests were used for pairwise comparisons, while one‐way ANOVA was used for multiple comparisons. All the graphs were drawn using GraphPad Prism 8.0.1 software.

## AUTHOR CONTRIBUTIONS

Weidong Zhang, Sanhong Liu, and Qun Wang performed the conceptualization, original draft, methodology, review and editing, funding acquisition, and supervision. Jianhua Xia and Mengting Xu carried out the experiments, generated the figures, and wrote the paper. Hongmei Hu, Qing Zhang, Dianping Yu, Minchen Cai, Xiangxin Geng, Hongwei Zhang, Yanyan Zhang, Mengmeng Guo, Dong Lu, Hanchi Xu, Linyang Li, and Xing Zhang participated in part of the experiments. All authors have read and approved the final manuscript.

## CONFLICT OF INTEREST STATEMENT

The authors declare no conflict of interest.

## ETHICS STATEMENT

All animal experiments were approved by the Ethics Committee of the Department of Laboratory Animal Science at Shanghai University of Traditional Chinese Medicine (SUTCM): Approval No: PZSHUTCM2306050010; PZSHUTCM2302080001.

## Supporting information

Supporting Information

## Data Availability

All data are available from the corresponding authors upon request.
