# Supplementary material for: 5,7,4′‐Trimethoxyflavone triggers cancer cell PD‐L1 ubiquitin–proteasome degradation and facilitates antitumor immunity by targeting HRD1
Source: MedComm (2020). 2024 Jun 27;5(7):e611. doi: 10.1002/mco2.611 (PMC11208742; doi:10.1002/mco2.611)
Supplement: Supplementary file 1 — Supporting Information [file MCO2-5-e611-s001.doc]

**5,7,4'-trimethoxyflavone triggers cancer cell PD-L1 ubiquitin-proteasome degradation and facilitates antitumor immunity by targeting HRD1**

Jianhua Xia^1#^, Mengting Xu^1#^, Hongmei Hu^1^, Qing Zhang^1^, Dianping Yu^1^, Minchen Cai^1^, Xiangxin Geng^1^, Hongwei Zhang^1^, Yanyan Zhang^1^, Mengmeng Guo^1^, Dong Lu^1^, Hanchi Xu^1^, Linyang Li^1^, Xing Zhang^1^, Qun Wang^1*^, Sanhong Liu^1*^, Weidong Zhang^1,2,3,4*^

^1^ Shanghai Frontiers Science Center of TCM Chemical Biology, Institute of Interdisciplinary Integrative Medicine Research, Shanghai University of Traditional Chinese Medicine, Shanghai, China

^2^ Department of Phytochemistry, School of Pharmacy, Second Military Medical University, Shanghai, China

^3^ Institute of Medicinal Plant Development, Chinese Academy of Medical Sciences and Peking Union Medical College, Beijing, China

^4^ The Research Center for Traditional Chinese Medicine, Shanghai Institute of Infectious Diseases and Biosafety, Institute of Interdisciplinary Integrative Medicine Research, Shanghai University of Traditional Chinese Medicine, Shanghai, China

^#^ These authors contributed equally to this work.

***Corresponding authors:**

**Weidong Zhang, Ph.D., Professor**

Address: Institute of Interdisciplinary Integrative Medicine Research, Shanghai University of Traditional Chinese Medicine, Shanghai, 201203, China

E-mail: wdzhangy@hotmail.com

**Sanhong Liu, Ph.D., Professor**

Address: Institute of Interdisciplinary Integrative Medicine Research, Shanghai University of Traditional Chinese Medicine, Shanghai, 201203, China

E-mail: liush@shutcm.edu.cn

**Qun Wang, Ph.D., Associate Professor**

Address: Institute of Interdisciplinary Integrative Medicine Research, Shanghai University of Traditional Chinese Medicine, Shanghai, 201203, China

E-mail: Qunwang0523@163.com

**Supplementary information**

**1. Supplementary information of Figure**


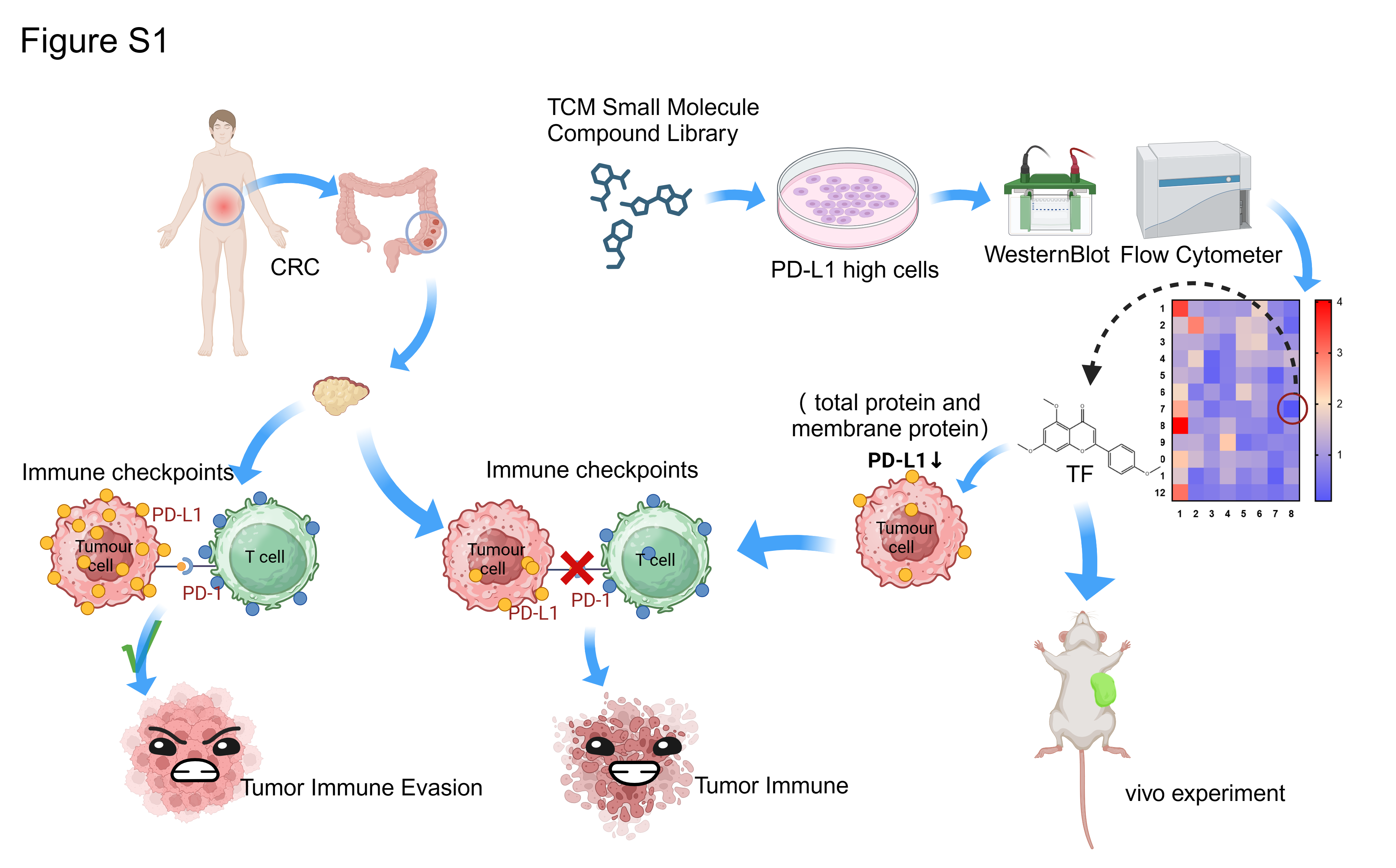


**Figure S1. Research background and flowchart of drug screening.** The heatmap shows the quantified immunoblotting results.


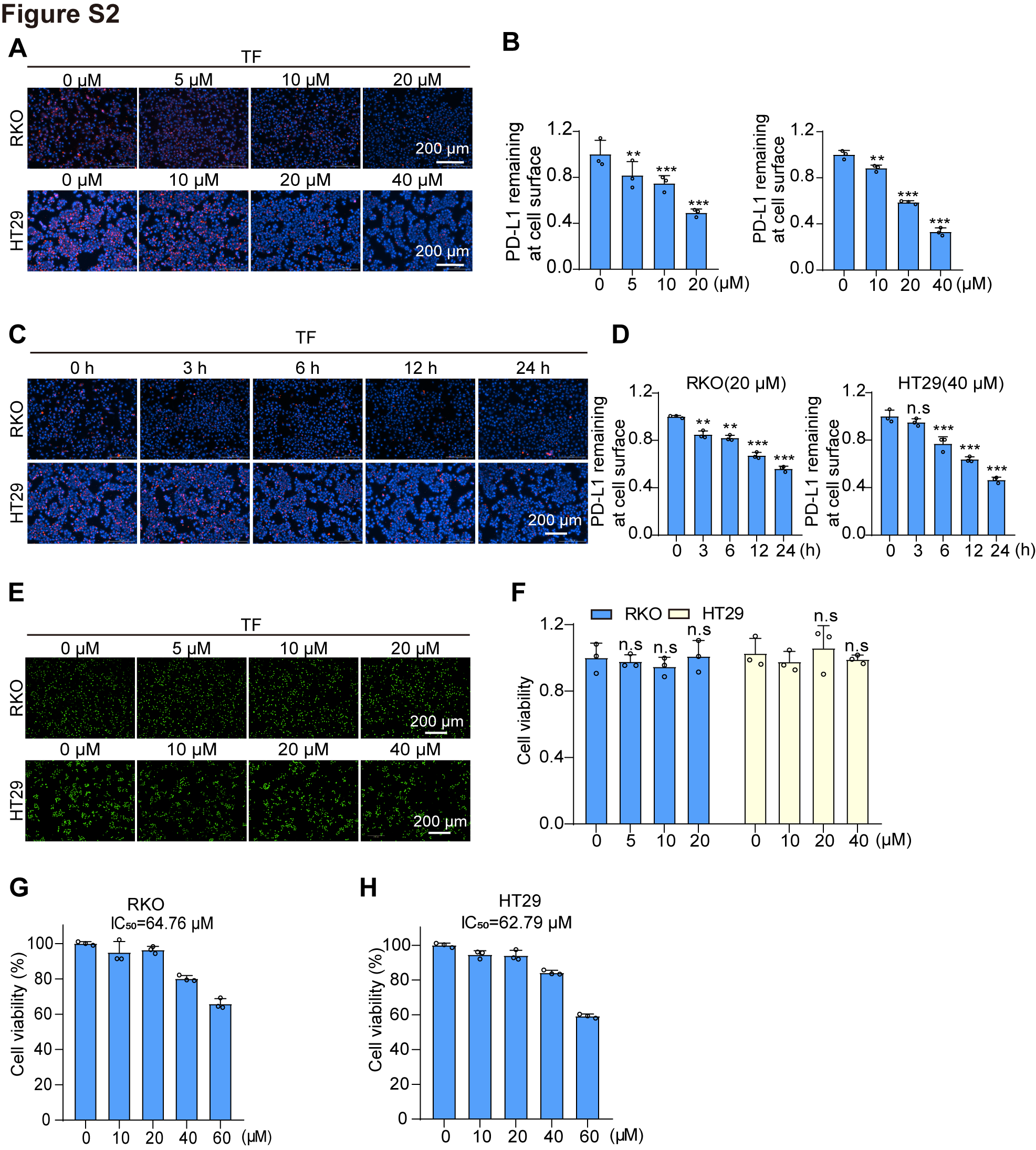


**Figure S2. TF degrades PD-L1 expression in Colorectal Cancer (CRC) cells with little cytotoxicity.** (A) Immunofluorescence was used to detect the expression of PD-L1 on the membranes of RKO and HT29 cells after 24 h of treatment with TF. Immunofluorescence staining showed PD-L1 labeling in red, and cell nuclei were labeled with DAPI. (Scale bar = 200 μm). (B) The results of the quantitative analysis of (A). (C) Immunofluorescence was used to detect the expression of PD-L1 on the membranes of RKO and HT29 cells treated with the indicated concentrations of TF for different times. Immunofluorescence staining showed PD-L1 labeling in red, and cell nuclei were labeled with DAPI. (Scale bar = 200 μm). (D) The results of the quantitative analysis of (C). (E) RKO and HT29 cells were treated with TF for 24 h. The effects of the drugs on the cells were detected using an EdU kit. (Scale bar = 200 μm). (F) The results of the quantitative analysis of (E). (G, H) RKO and HT29 cells were treated with different concentrations of TF for 24 h, and cell viability was determined by a CCK-8 assay. The data shown are the mean ± standard error of the mean (SEM). **p*<0.05, ***p*<0.01, ****p*<0.001.


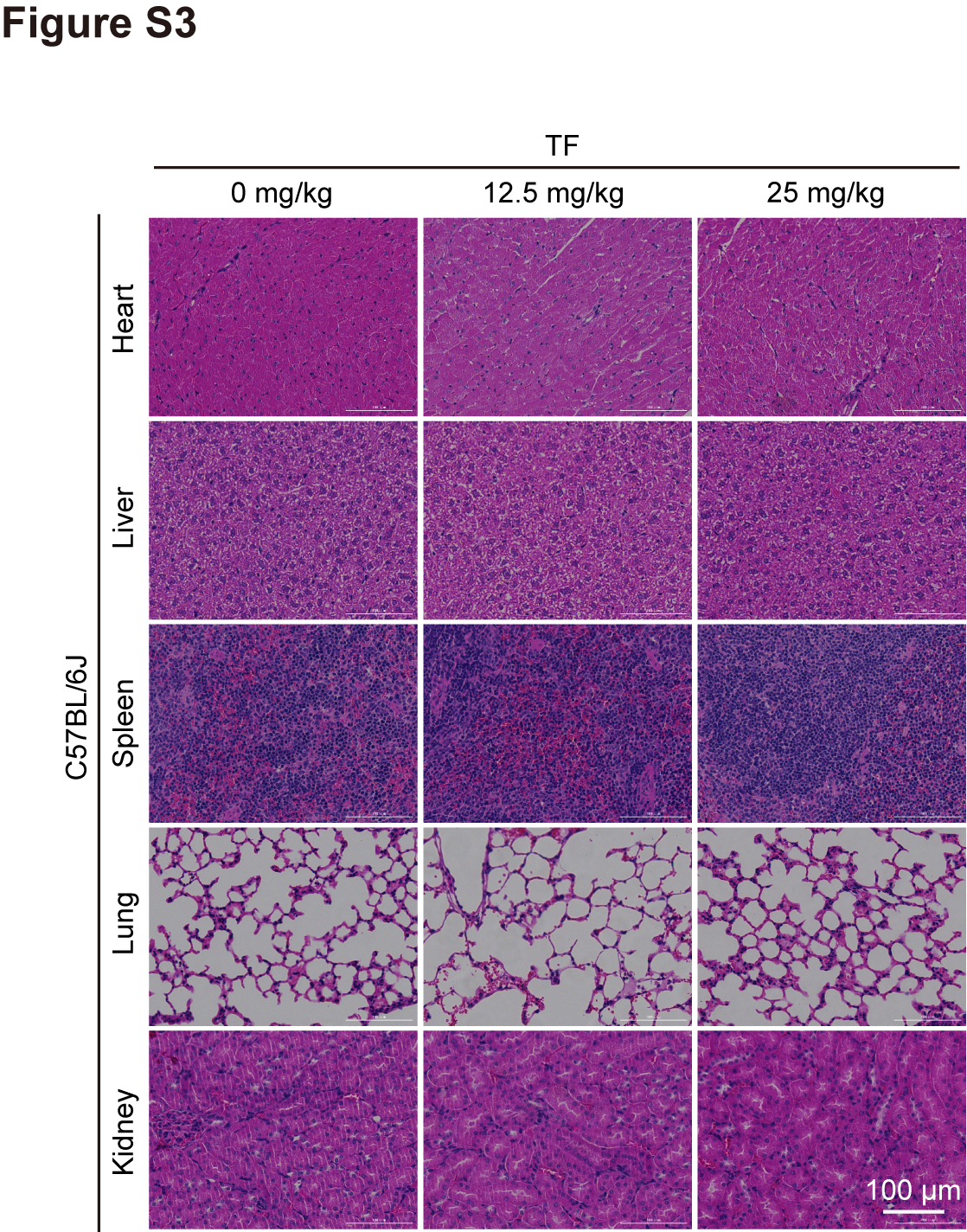


**Figure S3. TF has no significant toxic side effects in mice.** Hematoxylin-eosin staining of the main organs of C57BL/6J mice treated with saline or TF.


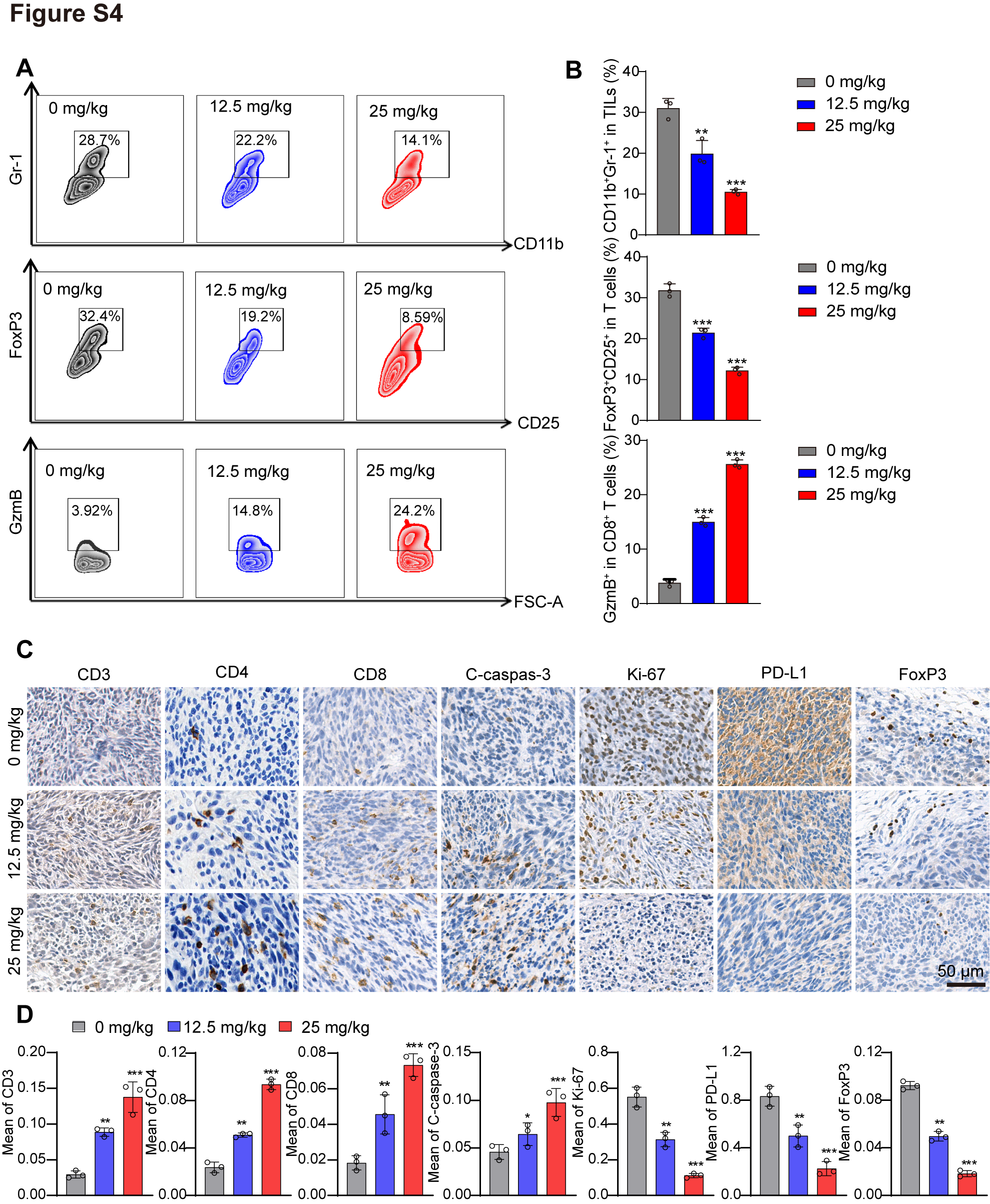


**Figure S4. TF enhances T cell killing capacity and mediates T cell-dependent antitumor effects *in vivo*.** (A) Flow cytometric analysis of GzmB^+^, Gr-1^+^CD11b^+^, and Foxp3^+^CD25^+^ cells in the saline, 0 mg/kg, 12.5 mg/kg, and 25 mg/kg TF treatment groups. (B) The results of the quantitative analysis of (A). (C) IHC staining results for CD3, CD4, CD8, C-caspas-3, Ki-67, PD-L1, and Foxp3. (Scale bar = 50 μm). (D) The results of the quantitative analysis of (C). The data shown are the mean ± standard error of the mean (SEM). **p*<0.05, ***p*<0.01, ****p*<0.001.


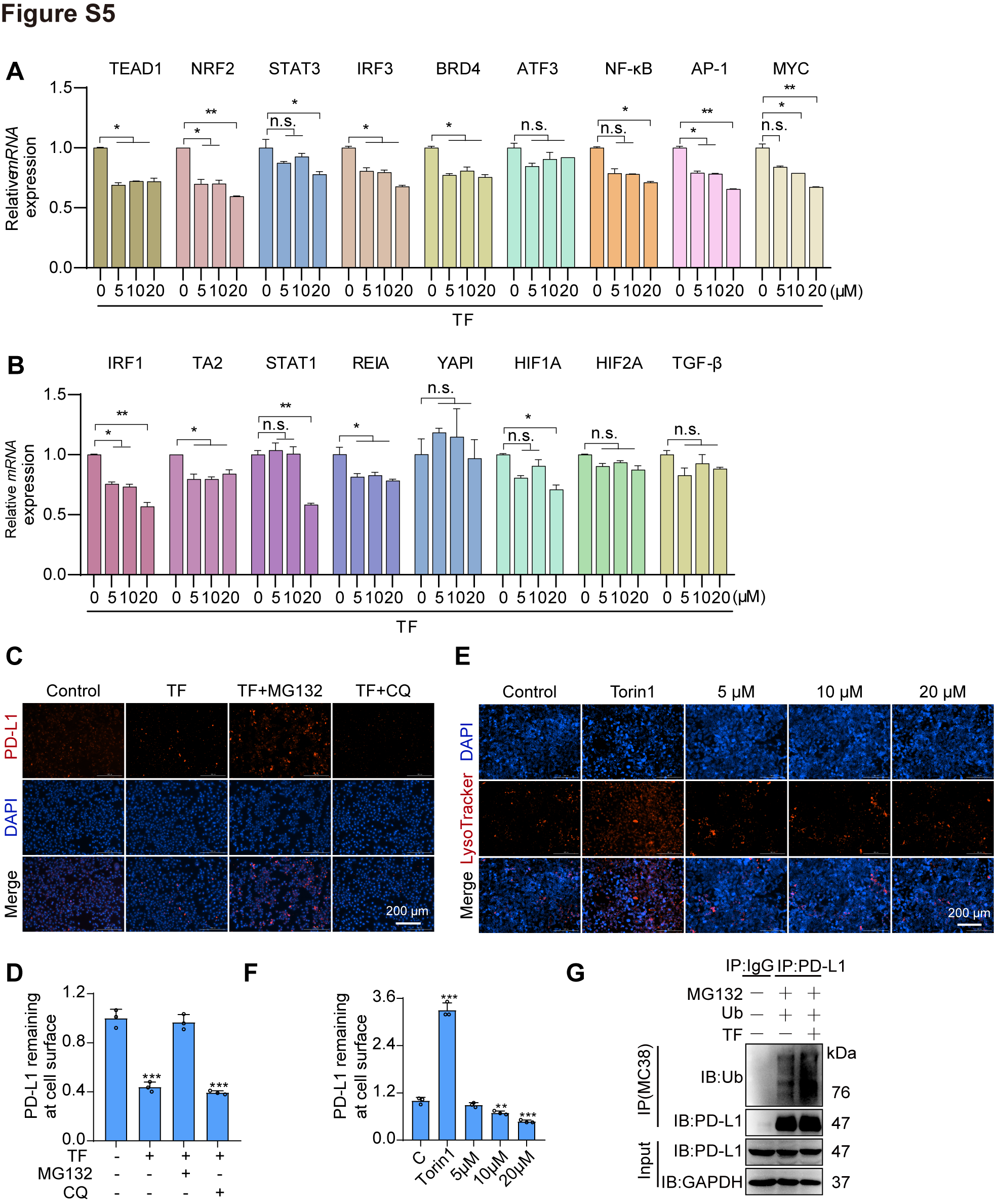


**Figure S5. TF mediates the degradation of PD-L1 via the ubiquitin proteasome pathway.** (A, B) Quantitative RT-PCR was used to analyze the mRNA levels of transcription factors regulating PD-L1 in RKO cells after 24 h of treatment with different concentrations of TF. (C) Immunofluorescence was used to detect the expression of PD-L1 on the membranes of RKO cells treated with TF (20 μM) in combination with MG132 (5 μM) or chloroquine (40 μM) for 24 hours. Immunofluorescence staining showed PD-L1 labeling in red, and cell nuclei were labeled with DAPI. (Scale bar = 200 μm). (D) The results of the quantitative analysis of (C). (E) RKO cells treated with TF (20 μM) or Torin1 (1 μM) for 24 h were stained with LysoTracker Red. (Scale bar = 200 μm). (F) The results of the quantitative analysis of (E). (G) Ub was overexpressed in MC38 cells to detect the TF-induced ubiquitination of PD-L1. Immunoprecipitation of ubiquitinated PD-L1 protein was performed using Flag bead pellets and immunoblotting with an anti-Ub antibody. The data shown are the mean ± standard error of the mean (SEM). **p*<0.05, ***p*<0.01, ****p*<0.001.


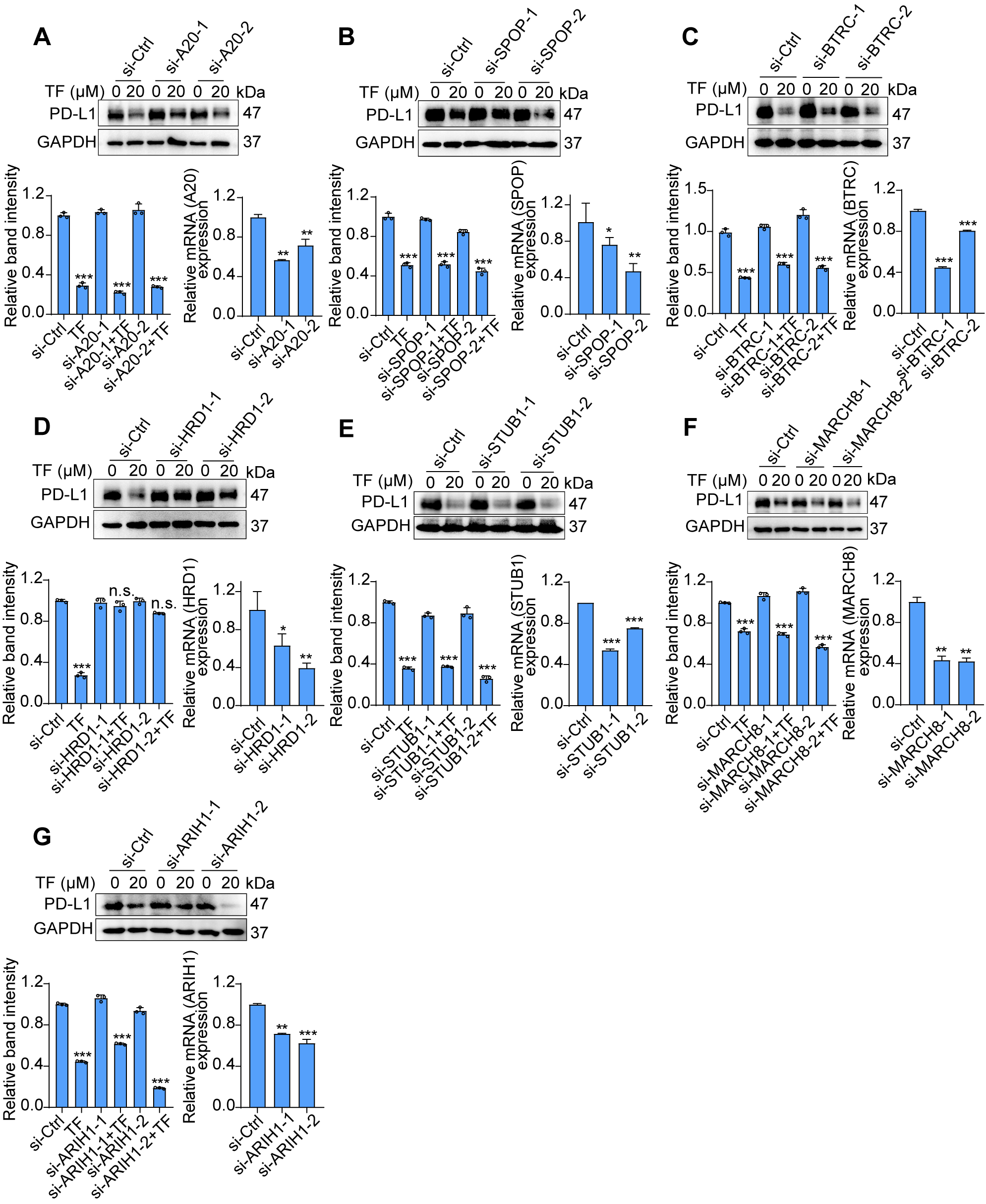


**Figure S6. Interference with HRD1 reverses PD-L1 degradation by TF.** (A-G) After transfection with negative control siRNA and siRNAs targeting A20, SPOP, BTRC, HRD1, STUB1, MARCH8, or ARIH1 for 48 h, RKO cells were treated with TF for 24 h, and PD-L1 levels were detected by protein immunoblotting. The results of the quantitative IB experiments and the knockdown efficiency of the siRNAs are shown below. The data shown are the mean ± standard error of the mean (SEM). **p*<0.05, ***p*<0.01, ****p*<0.001.


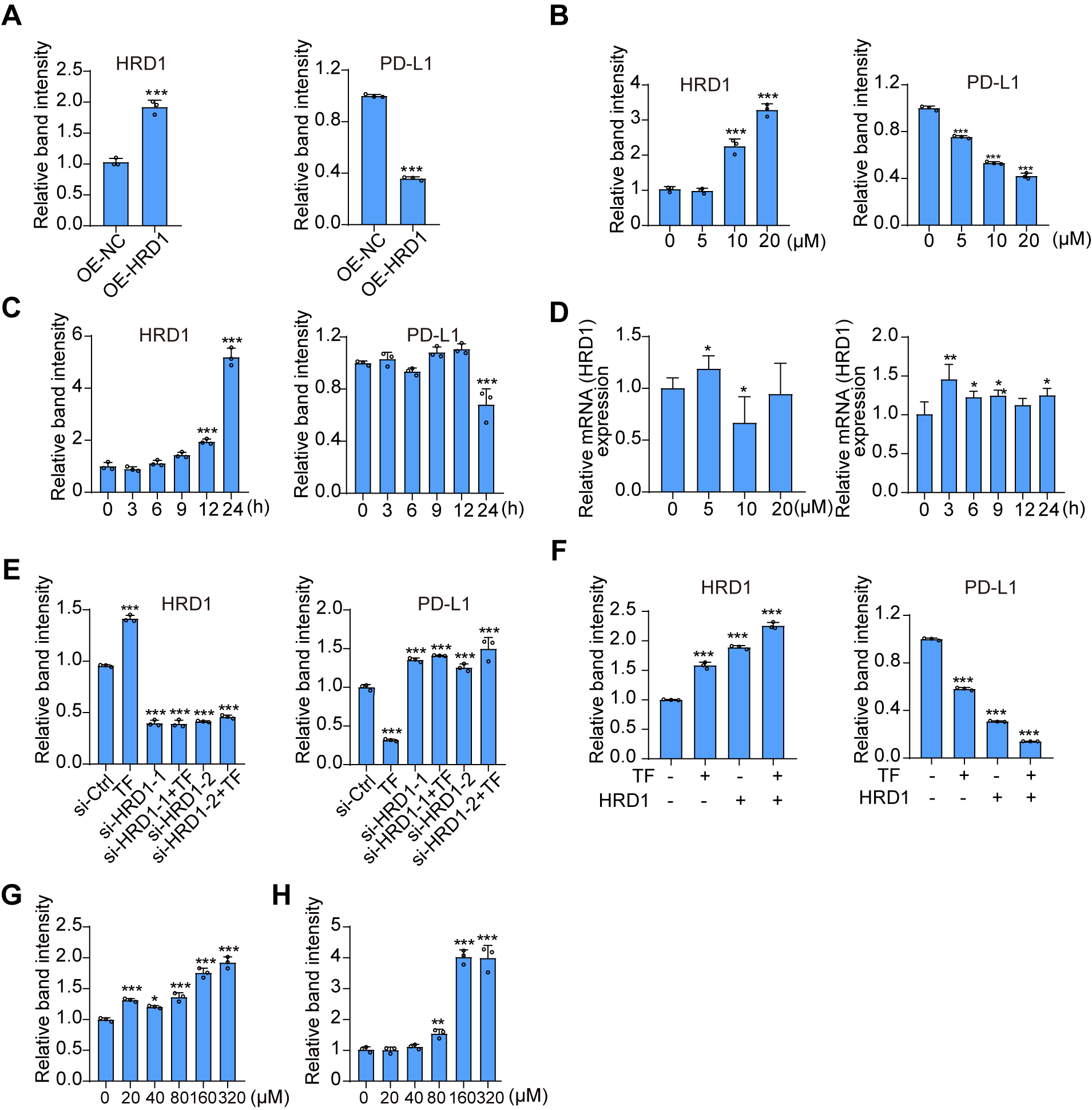


**Figure S7. Quantitative results of immunoblotting for HRD1 or PD-L1.** (A) Quantitative analysis of HRD1 and PD-L1 expression in cells overexpressing HRD1 by immunoblotting. (B, C) Quantitative results of HRD1 and PD-L1 levels detected by immunoblotting after treatment with different concentrations of TF for 24 h or the same concentration of TF for the indicated times. (D) Quantitative RT-PCR was used to analyze the HRD1 mRNA levels in RKO cells treated with different concentrations of TF for different times. (E) Quantitative results of protein blot analysis to detect PD-L1 and HRD1 protein expression in TF-treated RKO cells in the presence of siRNA targeting HRD1 or negative control (siRNA- Ctrl). (F) Quantitative results of protein blot analysis of PD-L1 and HRD1 protein expression in TF-treated RKO cells overexpressing HRD1 or negative control (OE-NC) cells. (G) Quantitative results of protein immunoblotting showing the stability of HRD1 at 62℃ with different concentrations of TF. (H) Quantitative results of protein immunoblotting for the stability of different concentrations of TF on HRD1 when the ratio of pronase to protein was 1:300. The data shown are the mean ± standard error of the mean (SEM). **p*<0.05, ***p*<0.01, ****p*<0.001.


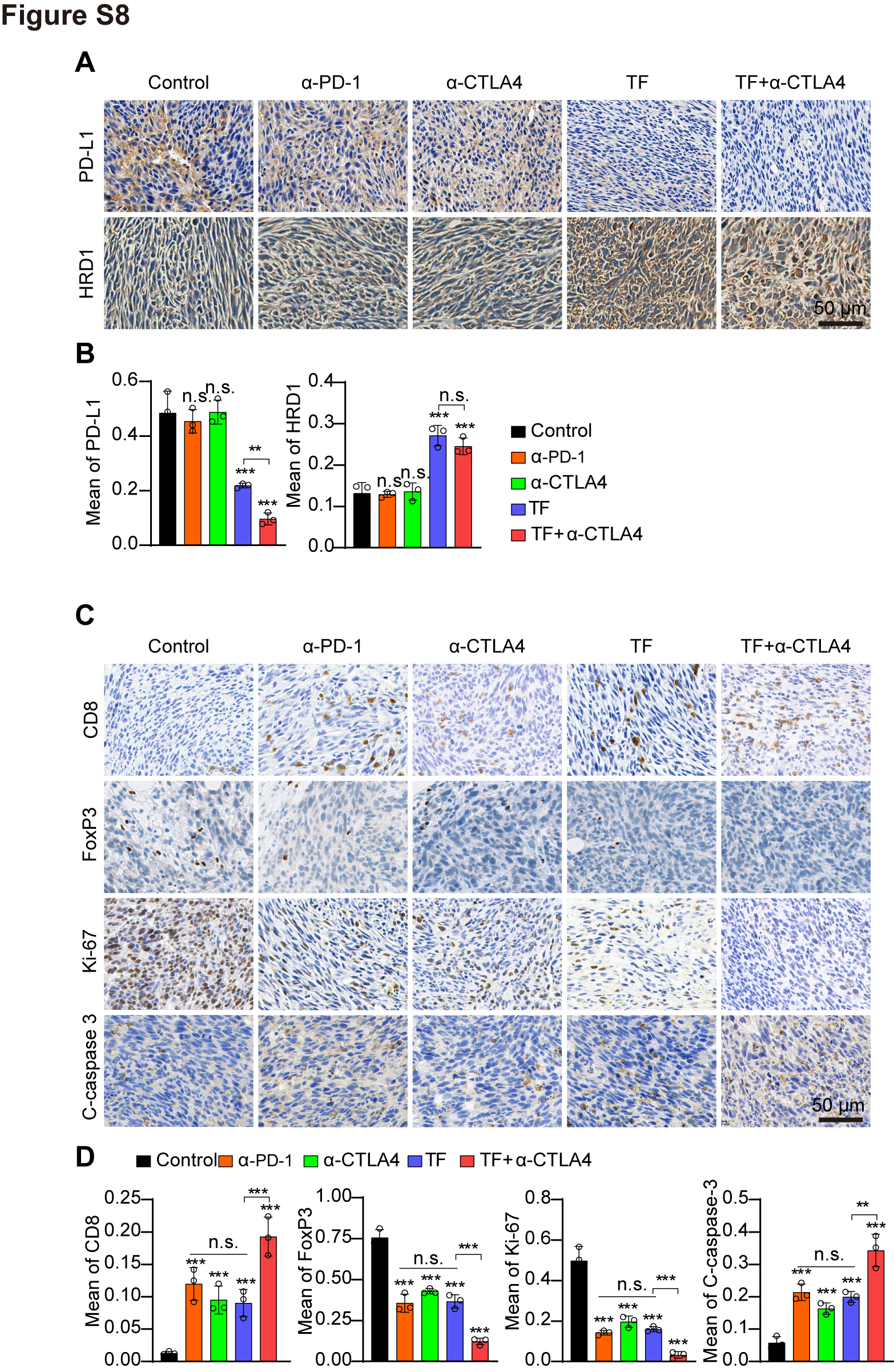


**Figure S8. TF is comparable to anti-PD-1 alone or anti-CTLA-4 alone for antitumor immunity.** (A) IHC staining results for PD-L1 and HRD1. (Scale bar = 50 μm). (B) The results of the quantitative analysis of (A). (C) IHC staining results for CD8, Foxp3, Ki-67, and C-caspas-3. (Scale bar = 50 μm). (D) The results of the quantitative analysis of (C). The data shown are the mean ± standard error of the mean (SEM). **p*<0.05, ***p*<0.01, ****p*<0.001.


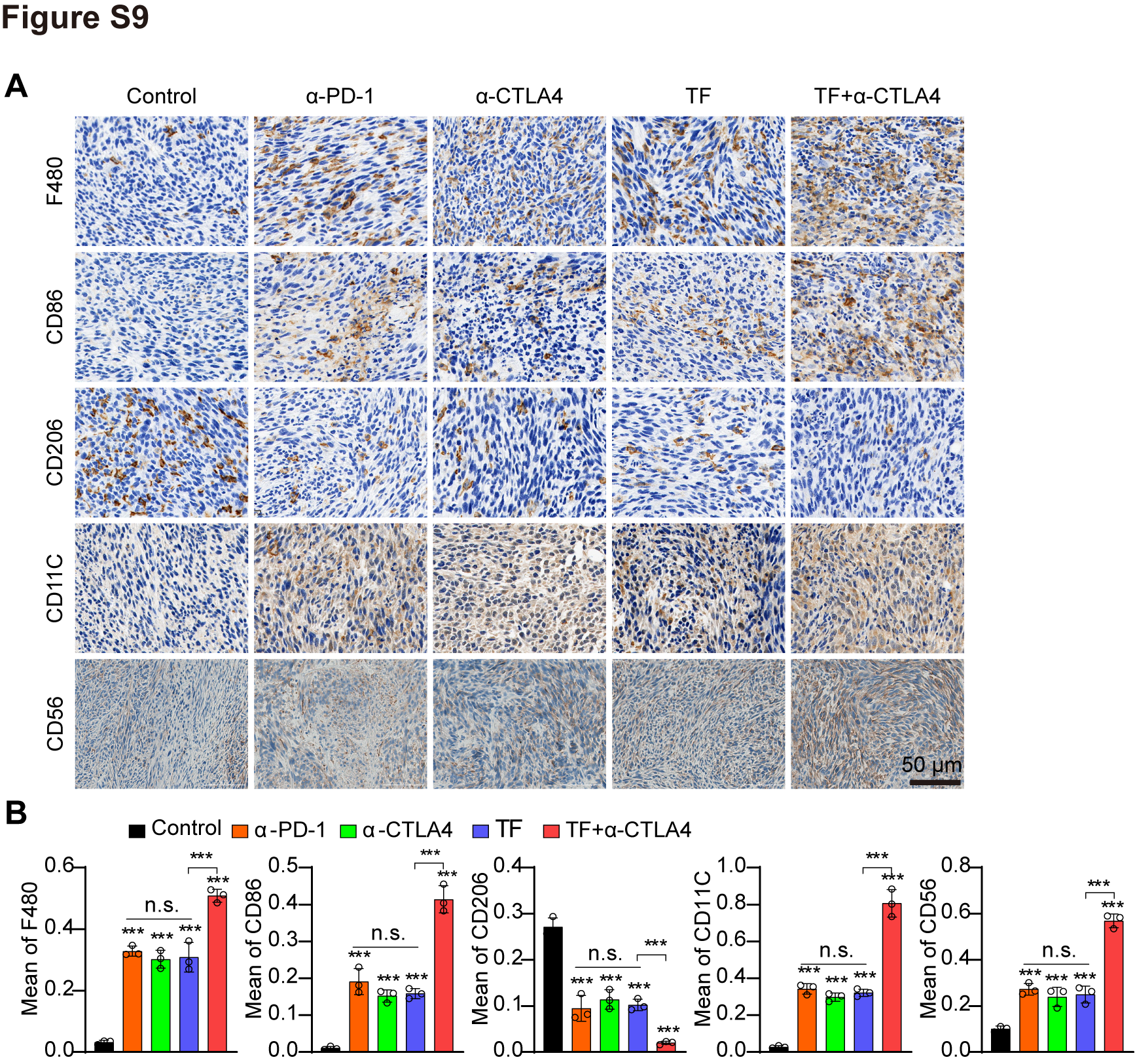


**Figure S9. Dendritic cells (DC), macrophages, and NK cells were detected by immunohistochemistry.** (A) IHC staining results for F480, CD86, CD206, CD11C, and CD56. (Scale bar = 50 μm). (B) The results of the quantitative analysis of (A). The data shown are the mean ± standard error of the mean (SEM). **p*<0.05, ***p*<0.01, ****p*<0.001.


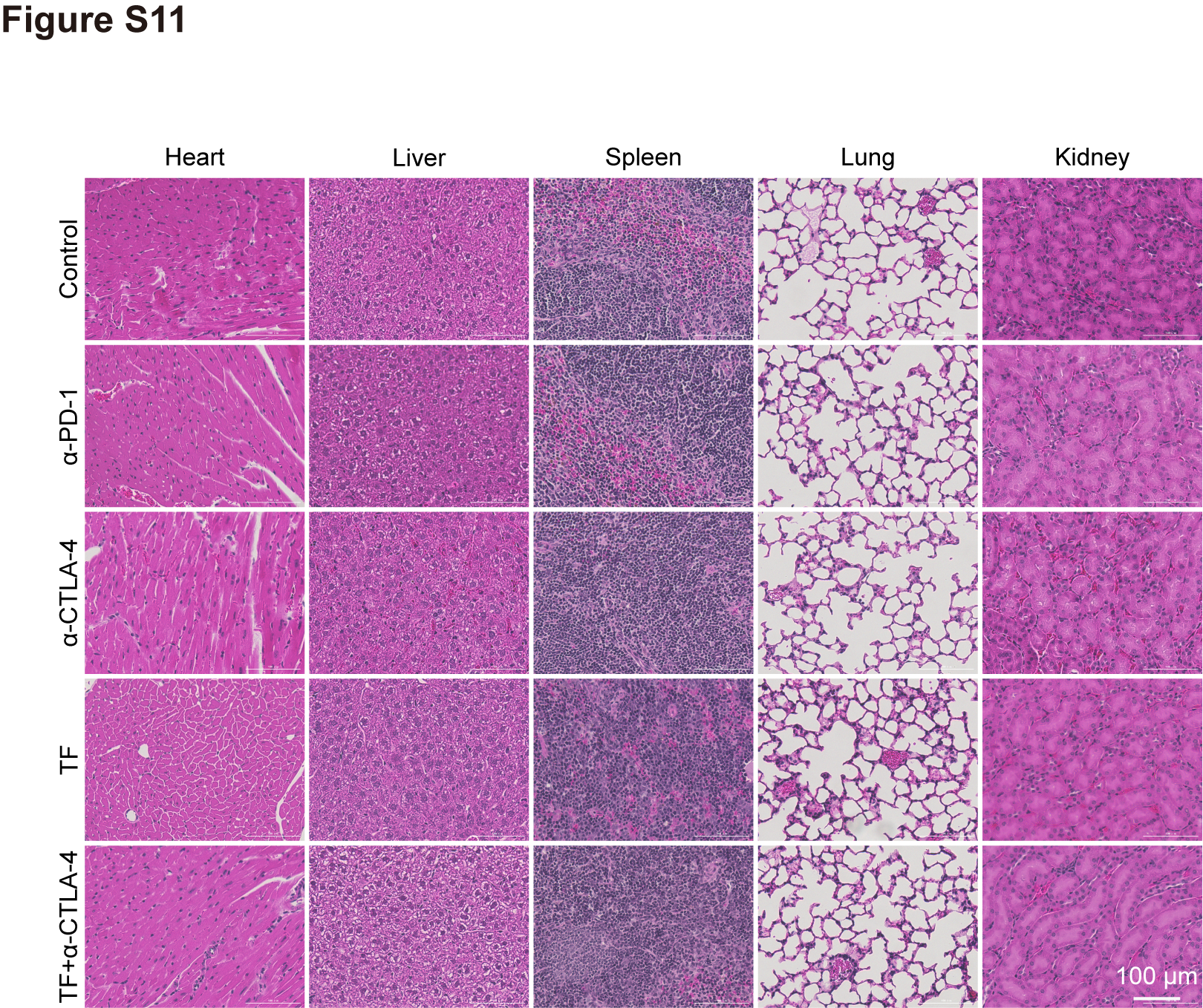


**Figure S10. TF and anti-CTLA-4 antibody combination had no significant toxic side effects on mice.** Hematoxylin-eosin staining of the main organs of C57BL/6J mice treated with saline or TF.

**2. Supplementary information of Table**

Supplementary Table 1. Antibody

| Name | Source | Catalog No. |
| --- | --- | --- |
| Anti-PD-L1 | Abcam | ab203103 |
| Anti-PD-L1 | Proteintech | 66248-1-Ig |
| Anti-Ubiquitin | Abcam | Ab7245 |
| Anti-HRD1 | Proteintech | 13473-1-AP |
| Anti-GAPDH | Proteintech | 60004-1-Ig |
| Anti-mouse PD-1 (CD279) | Invivogen | BE0146 |
| Anti-mouseCTLA4 (CD152) | Invivogen | BP0032 |
| PE anti-human CD274 | Biolegend | 329706 |
| PE anti-mouse CD274 | Biolegend | 124307 |
| PE anti-mouse CD25 | Biolegend | 101904 |
| Anti-Mouse CD4, PE-Cy7 | Multi Sciences | 70-F2100405/2-100 |
| Anti-Mouse Ly-6G(Gr-1), FITC | Multi Sciences | 70-F21LY6G01-100 |
| Anti-Mouse Foxp3, APC | Multi Sciences | 70-F21FP303-100 |
| Anti-Mouse CD3ε, APC-Cy7 | Multi Sciences | 70-F21003A06-100 |
| Anti-Mouse CD8α, PerCP-Cy5.5 | Multi Sciences | 70-F2100804-100 |
| Anti-Human/Mouse CD11b, mFluor 450 | Multi Sciences | 70-F41011b07-100 |
| Alexa Fluor® 700 anti-human/mouse Granzyme B Recombinant Antibody | Biolegend | 372222 |

**Supplementary Table 2. Primers for qRT‒PCR.**

| Name | Primers: forward primers (F), reverse primers (R) |
| --- | --- |
| PD-L1 | Forward: 5′-TCACTTGGTAATTCTGGGAGC-3′  Reverse: 5′-CTTTGAGTTTGTATCTTGGATGCC-3′ |
| β-actin | Forward: 5′- AGTGACCAGGCAGAAGATGC-3′  Reverse: 5′- CACGTACTCCATCGCTGACA -3′ |
| HRD1 | Forward: 5’-TGCGTAACATCCACACACTG-3’  Reverse: 5’-AGGCTAAACCTTCTGCCTTCA-3’ |
| SPOP | Forward: 5’-CCTGGAGCGCTTAAAGGTCA-3’  Reverse: 5’-AAAGGGAACACAGTGACGCA-3’ |
| A20 | Forward: 5’-AGGCCAATCATTGTCATTTC-3’  Reverse: 5’-AGAACAATGGGGTATCTGTAG-3’ |
| ARIH1 | Forward: 5’-ACTTTGATGGAAACCTGGAGAA-3’  Reverse: 5’-GCCAGTGAAATACGAGTTAGGG-3’ |
| MARCH8 | Forward: 5′-AGTGACATTCCACGTCATTGC-3′  Reverse: 5′-GATCTCCTCAGCAGTACGGTC-3′ |
| BTRC | Forward: 5’-TGGCTCATCTGACAACACTATC-3’  Reverse: 5’-CGAATACAACGCACCAATTCC-3’ |
| STUB1 | Forward: 5′-TCAAGGAGCAGGGCAATCGTCT-3′  Reverse: 5′-GCATCTTCAGGTAGCACAAGGC-3′ |
| ATF3 | Forward: 5′-CCTCTGCGCTGGAATCAGTC-3′  Reverse: 5′-TTCTTTCTCGTCGCCTCTTTTT-3′ |
| NRF2 | Forward: 5′- TCCAGTCAGAAACCAGTGGAT-3′  Reverse: 5′- GAATGTCTGCGCCAAAAGCTG -3′ |
| IRF3 | Forward: 5’-AGAGGCTCGTGATGGTCAAG-3’  Reverse: 5’-AGGTCCACAGTATTCTCCAGG-3’ |
| BRD4 | Forward: 5’-ACCTCCAACCCTAACAAGCC-3’  Reverse: 5’-TTTCCATAGTGTCTTGAGCACC-3’ |
| STAT3 | Forward: 5’-CAGCAGCTTGACACACGGTA-3’  Reverse: 5’-AAACACCAAAGTGGCATGTGA-3’ |
| TEAD1 | Forward: 5’-ATGCCAACCATTCTTACAGTGAC-3’  Reverse: 5’-ACAGTTCCTTTAAGCCACCTTTC-3’ |
| AP-1 | Forward: 5′-GTGCCGAAAAAGGAAGCTGG-3′  Reverse: 5′-GCTGCGTTAGCATGAGTTGG-3′ |
| MYC | Forward: 5’-CCCTCCACTCGGAAGGACTA-3’  Reverse: 5’-GCTGGTGCATTTTCGGTTGT-3’ |
| IRF1 | Forward: 5’-CTGTGCGAGTGTACCGGATG-3’  Reverse: 5’-ATCCCCACATGACTTCCTCTT-3’ |
| STAT1 | Forward: 5′-ATCAGGCTCAGTCGGGGAATA-3′  Reverse: 5′-TGGTCTCGTGTTCTCTGTTCT-3′ |
| NF-κB | Forward: 5′-CCCATCCCATGGTGGACTAC-3′  Reverse: 5′-CACCATGTCCTTGGGTCCAG-3′ |

**Supplementary Table 3. siRNA sequences used for knocking down the indicated proteins**

| Name | Sequence |
| --- | --- |
| si-HRD1#1 | 5′-UGUCUGGCCUUCACCGUUU-3′ |
| si-HRD1#2 | 5′-CCAAGAGACUGCCCUGCAA-3′ |
| si-SPOP#1 | 5′-CACAAGGCUAUCUUAGCAGCU-3′ |
| si-SPOP#2 | 5′-CUCCUACAUGUGGACCAUCAA-3′ |
| si-A20#1 | 5’-CCGAGCTGTTCCACTTGTTAA-3’ |
| si-A20#2 | 5’-CAGATGTATGGCTAACCGGAA-3’ |
| si-ARIH1#1 | 5′-CGAGAUAUUUCCCAAGAUUUU-3’ |
| si-ARIH1#2 | 5′-CCAUGUUGUUAAAGUCCAAUA-3’ |
| si-BTRC#1 | 5′-GCGUUGUAUUCGAUUUGAUAA-3′ |
| si-BTRC#2 | 5′-GCUGAACUUGUGUGCAAGGAA-3′ |
| si-MARCH8#1 | 5’-GGACATTTCATGAGT CATT-3’ |
| si-MARCH8#2 | 5’-GGAAGAGACTCAAGGCCTA-3’ |
| si-PD-L1#1 | 5'-TCAATTGTCATATTGCTAC-3' |
| si-PD-L1#2 | 5'-TTGACTCCATCTTTCTTCA-3' |
| si-STUB1#1 | 5′-GCAGUCUGUGAAGGCGCACUU-3′ |
| si-STUB2#2 | 5′-CCCAAGUUCUGCUGUUGGACU-3′ |

**3. Supplementary information for the Materials and Methods**

**3.1 Cell**

Human colorectal carcinoma cell lines (RKO and HT29) and a mouse colorectal carcinoma cell line (MC38) were acquired from the Shanghai Institute of Cell Biology, Chinese Academy of Sciences (Shanghai, China). Additionally, Jurkat cells were graciously provided by Kongming Wu's research group at the Department of Oncology, Tongji Hospital of Tongji Medical College, Huazhong University of Science and Technology, Wuhan, China. RKO cells were maintained in MEM (Meilunbio, Dalian, China), HT29 cells were maintained in McCoy’s 5A medium (Meilunbio, Dalian, China), MC38 cells were maintained in DMEM (Meilunbio, Dalian, China), and Jurkat cells were maintained in RIPM 1640 medium (Meilunbio, Dalian, China). The culture medium for all cell lines included 10% fetal bovine serum (Biological Industries, Cromwell, CT, USA), along with 100 mg/mL streptomycin and 100 U/mL penicillin. The cell cultures were incubated in a humidified atmosphere containing 5% CO_2_ at 37°C.

**3.2 Western blotting and immunoprecipitation**

After processing, the cells were lysed with RIPA lysis buffer (Beyotime, Haimen, China) (containing 1% protein inhibitor), and the protein concentration was determined using a BCA protein assay kit (Beyotime, Haimen, China). Subsequently, equal amounts of proteins were separated by SDS‒PAGE and transferred to PVDF membranes. After blocking with 5% skim milk, the membrane was incubated with a specific primary antibody at 4°C overnight. After the membrane was washed, it was incubated with the secondary antibody for 1 hour at room temperature. Scanning was performed using a Bio-Rad imaging system. The intensity of the bands was quantified using ImageJ software.

To analyze protein ubiquitination by immunoprecipitation (IP), transfected and treated cells were collected, proteins were lysed using IP lysis buffer (Beyotime, Haimen, China), and the cell lysates were immunoprecipitated by overnight incubation with an anti-PD-L1 antibody at 4°C, followed by pull-down with 30 μL of protein A/G beads at 4°C for 3 hours to capture the antibody-bound PD-L1. The beads were washed 5 times with lysis buffer before being subjected to immunoblotting. Subsequently, equal amounts of proteins were separated via SDS‒PAGE and transferred to PVDF membranes. Following blocking with 5% skim milk, the membrane was incubated with anti-Ub overnight at 4°C and then washed and incubated with a secondary antibody at room temperature for 1 hour. Scans were obtained using a Bio-Rad imaging system.

**3.3 RNA extraction and real-time PCR analysis**

Cells were lysed, and RNA was extracted using RNAiso-Plus (Takara, Shiga, Japan). Two milligrams of RNA was reverse transcribed to cDNA using the Prime Script RT Kit (Takara, Shiga, Japan). Two milligrams of RNA was then subjected to quantitative PCR using SYBR Green (Roche, Mannheim, Germany) and a LightCycler 96 instrument (Roche, Basel, Switzerland). β-actin was used as the reference gene. A LightCycler 96 instrument (Roche, Basel, Switzerland) was used for quantitative PCR, with β-actin serving as the reference gene. The specific primers used are listed in Table S2 of the Supporting Information.

**3.4 Transfection**

GenePharma (Shanghai, China) provided the small interfering RNAs (siRNAs) used for gene knockdown. The control used was a negative control (NC). The siRNA duplexes were transfected using a Liposome 2000 (Invitrogen, Carlsbad, CA). The cell culture medium was replaced with complete medium after 8 h of transfection, after which the cells were cultured for 48 h and subsequently treated with drugs for 24 h. The relevant siRNA sequences are shown in Supplementary Table S3. The plasmids used for transfection were pcDNA3.1-Ub and VP048 pCMV-MCS-3*Flag-SYVN1, which were purchased from GenePharma (Shanghai, China), and the HRD1-Glu216 and HRD1-Lys191 mutants were generated by side-directed mutagenesis PCR using platinum Pwo SuperYield DNA polymerase (NEB) according to the manufacturers’ instructions. All mutant plasmids were sequenced to confirm successful mutation and the absence of other unwanted mutations. The other steps and reagents used were the same as those used for siRNA transfection.

**3.5 Mice**

The experimental animals used in this study were 6- to 8-week-old female C57BL/6J mice and nude mice, which were purchased from Shanghai Jihui Laboratory Animal Breeding Co., Ltd. (Shanghai, China). All animal experiments were conducted in accordance with national and international standards and were approved by the Ethics Committee of the Department of Laboratory Animal Science at Shanghai University of Traditional Chinese Medicine (SUTCM).

A xenograft mouse tumor model was established by subcutaneous injection of 8×10^5^ MC38 cells. When the average tumor volume reached 50 mm^3^, the mice were randomly grouped, and appropriate treatment experiments were performed. The first batch of C57/6J mice was randomly divided into three groups and treated with PBS or 12.5 mg/kg or 25 mg/kg TF every day for 12 consecutive days; the body weights and tumor sizes of the mice were recorded every other day. Nude mice were randomly divided into two groups and treated daily with PBS or 25 mg/kg TF for 14 consecutive days; body weights and tumor volumes were recorded every other day. The second batch of C57/6J mice was randomly divided into five groups: the PBS group, the anti-PD-1 treatment group (100 μg/100 μL), the anti-CTLA4 treatment group (100 μg/100 μL), the TF group (25 mg/kg), and the combined treatment group of TF (25 mg/kg) and CTLA4 (100 μg/100 μL). PBS or TF was administered daily, and anti-PD-1 and anti-CTLA4 antibodies were injected every five days. In addition, the body weight and tumor volume of the mice were recorded every other day.

Tumor size was calculated using the following formula: volume (mm^3^) = length × (width^2^)/2. At the end of the experiment, the mice were killed, and the tumors and other major organs were collected.
